# The core planar cell polarity gene, *Vangl2*, directs adult corneal epithelial cell alignment and migration

**DOI:** 10.1098/rsos.160658

**Published:** 2016-10-19

**Authors:** Amy S. Findlay, D. Alessio Panzica, Petr Walczysko, Amy B. Holt, Deborah J. Henderson, John D. West, Ann M. Rajnicek, J. Martin Collinson

**Affiliations:** 1 Institute of Genetic Medicine, Newcastle University, Centre for Life, Newcastle upon Tyne NE1 3BZ, UK; 2 Genes and Development Group, Centre for Integrative Physiology, Clinical Sciences, University of Edinburgh Medical School, Hugh Robson Building, George Square, Edinburgh EH8 9XD, UK; 3 School of Medicine, Medical Sciences and Nutrition, University of Aberdeen, Institute of Medical Sciences, Aberdeen AB25 2ZD, UK

**Keywords:** cornea, epithelium, planar cell polarity, Vangl2, cell migration

## Abstract

This study shows that the core planar cell polarity (PCP) genes direct the aligned cell migration in the adult corneal epithelium, a stratified squamous epithelium on the outer surface of the vertebrate eye. Expression of multiple core PCP genes was demonstrated in the adult corneal epithelium. PCP components were manipulated genetically and pharmacologically in human and mouse corneal epithelial cells *in vivo* and *in vitro*. Knockdown of *VANGL2* reduced the directional component of migration of human corneal epithelial (HCE) cells without affecting speed. It was shown that signalling through PCP mediators, dishevelled, dishevelled-associated activator of morphogenesis and Rho-associated protein kinase directs the alignment of HCE cells by affecting cytoskeletal reorganization. Cells in which *VANGL2* was disrupted tended to misalign on grooved surfaces and migrate across, rather than parallel to the grooves. Adult corneal epithelial cells in which *Vangl2* had been conditionally deleted showed a reduced rate of wound-healing migration. Conditional deletion of *Vangl2* in the mouse corneal epithelium ablated the normal highly stereotyped patterns of centripetal cell migration *in vivo* from the periphery (limbus) to the centre of the cornea. Corneal opacity owing to chronic wounding is a major cause of degenerative blindness across the world, and this study shows that Vangl2 activity is required for directional corneal epithelial migration.

## 1. Introduction

The outermost layer of the vertebrate eye is a stratified squamous corneal epithelium that performs a barrier function essential to maintenance of corneal transparency and ocular health. It has an efficient wound-healing response: the basal layer of corneal epithelial cells is highly proliferative and may have stem cell activity [1]. However, stem cells also reside at the periphery of the cornea, the limbus, at the boundary between corneal and conjunctival epithelium (limbal epithelial stem cells, henceforth LESCs) [2]. Cells are constantly lost from the corneal epithelial surface owing to abrasion or injury and multiple studies have shown that there is continuous centripetal epithelial cell migration from the limbus to the centre of the cornea throughout adult life [3–5]. A homeostatic balance between LESC activity, migration of cells into the cornea from the limbus, proliferation of corneal epithelial cells, cell death and desquamation is required for corneal epithelial maintenance [6,7]. Studies using transgenic mice with mosaic reporter gene activity have shown that corneal epithelial cell migration is strikingly regular, long distance and can be disrupted by genetic mutation or physical injury [7,8]. Failure of ocular surface homeostasis and subsequent corneal opacity, caused by genetic abnormalities, injury or infection, is the most common cause of degenerative blindness in the developing world. It is essential to understand the mechanisms by which the cornea is maintained.

The molecular control of directionality of corneal epithelial cell migration is poorly understood. Chemical, physical and electrical guidance signals have been postulated [9–11]. In other systems, directional behaviour of epithelial cells in the plane of their basement membranes has been shown to derive from a non-canonical Wnt-related, planar cell polarity (PCP) pathway [12]. PCP was initially studied in *Drosophila* in highly organized tissues including eye and wing [12–14]. Planar polarity in invertebrate systems derives from interaction between transmembrane proteins frizzled and van gogh/strabismus (vang), asymmetrically localized to membranes on opposite sides of the cell, alongside interaction of other cadherin-like transmembrane proteins such as flamingo/CELSR, conferring patterns of epithelial directionality. The downstream PCP pathway comprises the two branches of the non-canonical Wnt-signalling cascade (reviewed in [15]). One branch signals downstream through the dishevelled (*dvl*) DEP domain to c-Jun N-terminal kinase (JNK). A second branch acts through dishevelled-associated activator of morphogenesis (DAAM) binding to the PDZ domain of dishevelled, initiating downstream signalling to RhoA and rho-associated protein kinase (ROCK) prompting planar-polarized cytoskeletal reorganization [15]. In *Drosophila*, clones of PCP-mutant cells in the cuticular epithelium can disrupt the polarity of adjacent non-mutant cells [14], but to date, there is no evidence for comparable non-autonomous signalling in vertebrates. A parallel PCP system (‘fatPCP’) mediated by cadherin-like proteins fat/dachsous may act to impose the directionality of fzPCP activity, representing a global patterning mechanism [16]. Vertebrates have multiple PCP gene homologues including genes encoding the van gogh homologue, vang-like 2 (*Vangl2*) and its putative binding partners, frizzled homologues Frizzled-3 (*Fzd3*) and Frizzled-6 (*Fzd6*), that have been shown to have multiple planar polarity functions during vertebrate embryogenesis and disease [17–19]. The roles of PCP pathways after embryogenesis in vertebrates are not well studied, although PCP activity has recently been implicated in metastatic and invasive behaviour of tumours [18,20]. In this study, it was hypothesized that PCP core proteins are required for directional cell migration within the corneal epithelium. It was shown for the first time that multiple PCP components are expressed in the adult corneal epithelium. Planar polarity components of the adult corneal epithelium were disrupted *in vitro* and *in vivo* by conditional knockout and knockdown of the core PCP genes, *Vangl2* and *Fzd6*. The study shows that disrupting Vangl2 activity confers a planar defect in an adult vertebrate system.

## 2. Results

### 2.1. Multiple core planar cell polarity components are expressed in the adult corneal epithelium

**Figure 1.**
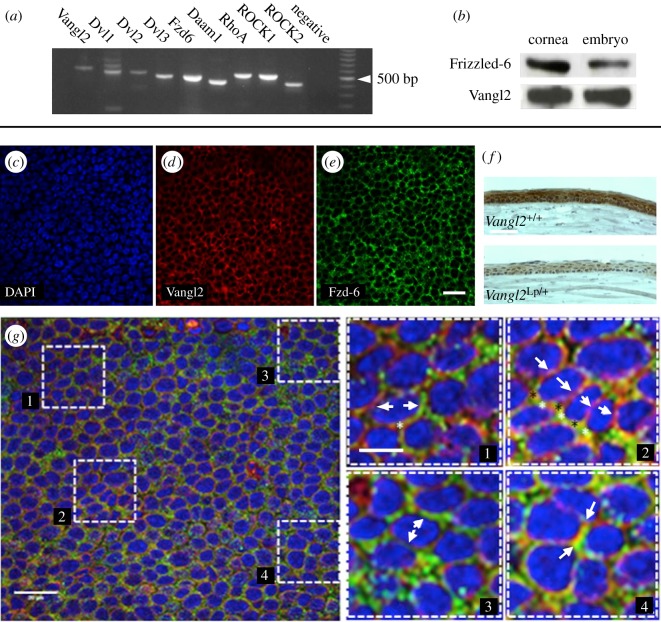
Localization of core PCP components to the adult corneal epithelium. (*a*) RT-PCR on cDNA prepared from adult mouse corneal epithelium shows the expression of core PCP components and pathway mediators (see also electronic supplementary material, figure S1). (*b*) Western blot from sodium dodecylsulfate polyacrylamide gel electrophoresis gel of protein lysate from adult mouse corneas and E18.5 mouse whole-embryos (as positive control), probed with antibodies against Frizzled-6 and Vangl2. (*c–e*) Frizzled-6 and Vangl2 immunohistochemistry on basal epithelial cells of flat-mounted adult mouse corneas. (*c*) DAPI nuclear stain (blue). (*d*) Vangl2 (red). (*e*) Fzd-6 (green). (*f*) Vangl2 immunohistochemistry on tissue sections of wild-type and *Vangl2^Lp/+^* littermates. Tissues and sections were processed and stained simultaneously. (*g*) Overlaid DAPI/Vangl2/Fzd-6 confocal images show (insets) areas of: 1, 3. Apparent polarized asymmetric localization of Fzd-6 and Vangl2 in single cells; 2. Apparently coordinated localization exclusively of Fzd-6 at lateral edges of several cells (parallel to the long axis of the cells), with punctate mutual exclusion of Fzd-6 and Vangl2 along the short axes; 4. Colocalization at the level of light microscope resolution. Scale bars, 20 µm (*e,g*).

Reverse transcription polymerase chain reaction (RT-PCR) showed for the first time that multiple core PCP pathway components are expressed in the adult mouse and in a human corneal epithelial (HCE) cell line (figure 1*a* and electronic supplementary material, figure S1 and table S1). This included not only the genes encoding core PCP transmembrane proteins such as Vangl2, Frizzled-3 (Fzd3) and Frizzled-6 (Fzd6), and flamingo homologue Celsr-1, but also intracellular intermediates dishevelled-1 (Dvl1), dishevelled-2 (Dvl2) and dishevelled-3 (Dvl3), DAAM1 and downstream mediators of signalling, RhoA, protein tyrosine kinase 7 (Ptk7), ROCK1 and ROCK2. Protocadherins FAT4 and Dachsous-1 were also found to be expressed in the adult corneal epithelium (electronic supplementary material, figure S2 and table S2).

Western blot and immunohistochemical analysis on adult mouse eyes confirmed that the core PCP proteins Vangl2 and Fzd6 were present and localized to all cells of the corneal epithelium (figure 1*b–e*). The corneal epithelium of adult *Vangl2^Lp/+^* heterozygotes (loop tail mice, heterozygous for an inactivating mutation in *Vangl2*) was superficially normal, but immunostaining for Vangl2 was weaker than in wild-type (figure 1*f*). Confocal analysis of flat-mounted mouse corneal epithelia immunostained for Vangl2 and its potential binding partner, Frizzled-6, was performed. Apparent colocalization of the two proteins around cell–cell boundaries was observed, but areas of apparent mutual exclusion were also recorded in z-stack images (figure 1*g*).

### 2.2. Knockdown of Vangl2 inhibits planar directionality of corneal epithelial cell migration

**Figure 2.**
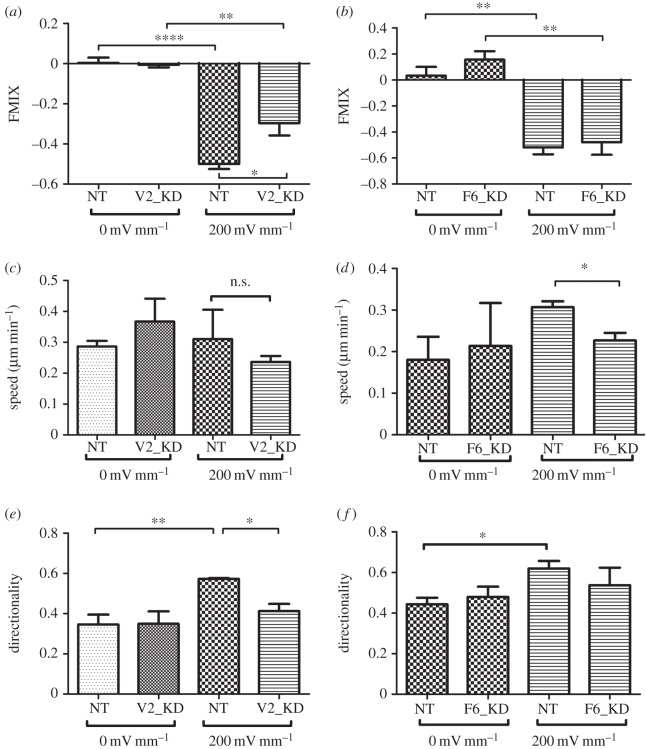
Knockdown of Vangl2 but not Fzd6 inhibits polarized human corneal epithelial (HCE) cell migration. Movement of HCE cells was monitored *in vitro* in control conditions (0 mV mm^−1^) or in presence of a physiological electric field (200 mV mm^−1^). Cells were transfected with nonsense siRNA (NT) as controls, siRNA targeting Vangl2 (V2_KD) or Fzd6 (F6_KD). (*a,b*) Cathodal migration expressed as FMIX, the mean ratio of distance moved on the *x*-axis towards or away from the cathode, divided by the total distance moved by the cell, with FMIX = −1 representing perfect cathodal migration. In absence of a field, cells migrated randomly (FMIX approx. 0). On application of the electric field, NT control cells migrated cathodally (FMIX < 0). Knockdown of Vangl2 (*a*) but not Fzd6 (*b*) caused significant decrease in the cathodal migratory response (*p* = 0.04). (*c,d*) Knockdown of Fzd6 (*d*) but not Vangl2 (*c*) caused a significant decrease in cell migration speed in the applied electric field (*p* = 0.03). (*e,f*) Mean directionality (the ratio of straight line distance migrated by each cell divided by the total length of its migration track) increased significantly in NT control cells upon application of the electric field, indicating more directed migration. However, knockdown of Vangl2 (*e*) but not Fzd6 (*f*) significantly reduced the increase in directionality ( *p* = 0.01). Together, the data show that knockdown of Vangl2 levels decreases polarized cell migration not by affecting migration speed but by reducing the cell's ability to stop wandering randomly and migrate directionally in response to the guidance cue.

Multiple studies by this and other groups have shown that corneal epithelial cells migrate cathodally in an applied electric field of a magnitude similar to that which they experience *in vivo* after wounding [21,22]. An *in vitro* cell migration assay was used to determine whether PCP pathways were required for directed cell movement. HCE cells were targeted with siRNAs against *VANGL2* or *FZD6*, leading to a mean 68% and 65% reduction in mRNA levels compared with those transfected with nonsense non-targeting siRNA (electronic supplementary material, figures S3*a* and S4*a*). Immunocytochemistry and Western blot confirmed the rates of successful transfection as high as 90% and subsequent knockdown of the proteins (electronic supplementary material, figures S3*b,c* and S4*b,c*). When an electric field is applied, corneal epithelial cells normally show a robust migration towards the cathode. VANGL2-knockdown cells did not migrate as efficiently towards the cathode as control cells transfected with a nonsense siRNA when a physiological electric field of 200 mV mm^−1^ was applied to the cell culture (figure 2 and electronic supplementary material, figure S5). Cathodal migration was expressed as the forward migration index (FMIX) as described in Materials and methods. Briefly, the FMIX for a cell is a ratio of the displacement towards the cathode during the experiment divided by the total distance travelled by the wandering cell, with FMIX of −1 representing direct migration to the cathode and FMIX of +1 representing perfect anodal migration. FMIX of 0 represents random migration (expressed as a mean of all cells in a culture). Both nonsense siRNA cell (NT) and VANGL2-knockdown cells (V2_KD) migrated randomly on tissue culture plastic in the absence of an electric field (FMIX ± s.e.m.: NT = −0.003 ± 0.026; *V2*_KD = −0.006 ± 0.012; *n* = 3 independent experiments). Nonsense siRNA cells migrated cathodally in the applied electric field (FMIX = −0.4825 ± 0.025; *n* = 3), but the VANGL2-knockdown cells showed a significantly reduced, i.e. less negative, response (FMIX = −0.33 ± 0.055; *n* = 3; *t*-test: *p* = 0.04; figure 2*a*). There was no significant difference in migration speed between control and experimental cells (figure 2*c*). Instead the directness of migration of the cells in the electric field (‘directionality’ measured as the ratio of the total displacement of the cell during the experiment divided by the total length of the track of its migration, with directionality of 1 representing straight line migration) was different. For control nonsense siRNA cells in the electric field, directionality was a very consistent 0.57 ± 0.003. For Vangl2-knockdown cells, directionality was significantly lower (0.41 ± 0.034; *n* = 3 independent experiments; *p* = 0.010) indicating a more wandering, less directed migration (figure 2*e*) and equivalent to their directionality in absence of a field. These data showed that knockdown of Vangl2 does not stop cells recognizing the applied guidance cue and changing direction appropriately, but reduces the efficiency of directional migration in response to the physiological planar cue, at least in part by causing the cells to migrate on a more wandering track (i.e. decreased directionality). In contrast to nonsense siRNA control cells, the directionality of Vangl2-knockdown cells did not increase upon application of the electric field.

Knockdown of FRIZZLED-6 using siRNA (electronic supplementary material, figure S4) decreased the speed of cell migration in knockdown (F6_KD) cells in an electric field (NT = 0.031 µm min^−1^ ± 0.014; F6_KD = 0.23 µm min^−1^ ± 0.018; *n* = 3 independent experiments; *p* *=* 0.03). However, the knockdown did not by itself lead to a defect of cathodal migration in the 200 mV mm^−1^ EF (FMIX ± s.e.m.: NT = −0.52 ± 0.05; *F6_KD* = −0.48 ± 0.09; *n* = 3; *p* > 0.05) or affect the directionality (figure 2*b,d,f*).

### 2.3. Vangl2 is required for normal corneal epithelial wound repair

**Figure 3.**
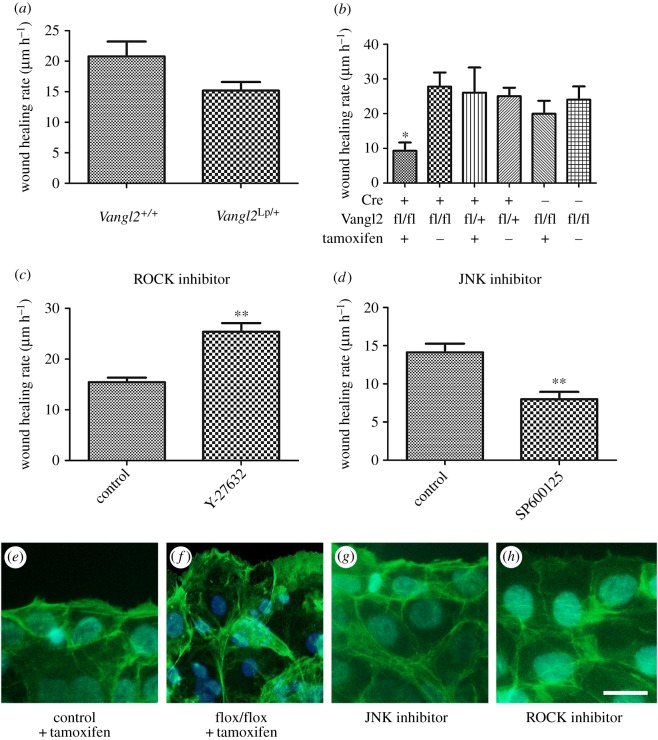
Deletion of Vangl2 or pharmacological disruption of PCP mediators causes a wound-healing defect in corneal epithelial cells. (*a*) Wound-healing rate of corneal epithelial cells from adult wild-type and *Vangl2^Lp/+^* littermates. *Vangl2^Lp/+^* cells trended downwards but the difference was not significant. (*b*) Conditional deletion of *Vangl2* in corneal epithelial cells reduced wound-healing migration to 50–30% of that shown by controls (*p = 0.023). (*c,d*) Pharmacological treatment of corneal epithelial cells with ROCK inhibitor, Y-27632, (*c*) caused a significant increase in wound-healing rate (*p* = 0.001), whereas treatment with JNK inhibitor, SP600125, (*d*) caused a significant decrease in wound-healing rate (*p* = 0.001). (*e–h*) Alexa488-phalloidin labelling of the actin cytoskeleton of actively healing corneal epithelial cells. Controls (*e*), *CAGG-ERTM^+^ Vangl2^flox/flox^* tamoxifen-treated cells (*f*) and JNK-inhibited cells (*g*) showed a similar concentration of actin network and focal adhesions in the leading edge cells and behind the wound, in contrast to ROCK-inhibited cells (*h*). Scale bar, 15 µm.

Scratch-wounding of corneal epithelia was performed *in vitro* as described in the Materials and methods section in order to determine whether Vangl2 was required for wound-healing cell migration. Monolayers of mouse corneal epithelial cells from adult *Vangl2^Lp/+^* mice and their wild-type littermates were cultured and scratch-wounded as described in [23]. Wound-healing rate trended downwards in *Vangl2^Lp/+^* corneal epithelia, but the difference was not significant (mean rate µm h^−1^ ± s.e.m.; *Vangl2^Lp/+^* 15.18 ± 1.41; *Vangl2^+/+^* 20.78 ± 2.44; *t*-test: *p* = 0.0849; *n* = 6 wild-type; seven *Vangl2^Lp/+^* mice; figure 3*a*). Because the morphology and wound-healing rate of heterozygous *Vangl2^Lp/+^*corneal epithelia were normal, or nearly so, and *Vangl2^Lp/Lp^* homozygous null mice die *in utero*, Cre/*loxP* technology was used to generate conditional knockouts.

*CAGG-CreERTM^Tg/−^* mice (*Tg*(*CAG-cre/Esr1*)) which constitutively express a transgene encoding tamoxifen-inducible Cre recombinase were bred to *Vangl2^flox/flox^* mice [24] and the *CAGG-CreERTM^Tg/−^ Vangl2^flox/+^* progeny backcrossed to *Vangl2^flox/flox^* to yield *CAGG-CreERTM^Tg/−^ Vangl2^flox/flox^* conditional knockouts and *CAGG-CreERTM^Tg/−^ Vangl2^flox/+^* controls. When monolayers of corneal epithelial cells were cultured from these mice, it was found that addition of 10 nM 4-OH tamoxifen to the medium caused nuclear relocalization of the CreERTM within 24 h, knocking out *Vangl2* and efficiently removed Vangl2 protein from the cells within 48 h (electronic supplementary material, figure S6).

To determine whether a planar migration defect was detectable in *Vangl2*-null corneal epithelial cells, scratch-wound assays were performed. Monolayers were cultured from adult *CAGG-CreERTM^Tg/−^ Vangl2^flox/flox^* and *CAGG-CreERTM^Tg/−^ Vangl2^flox/+^* mice. In the absence of tamoxifen, *CAGG-CreERTM^Tg/−^ Vangl2^flox/+^* and *CAGG-CreERTM^Tg/−^ Vangl2^flox/flox^* cells showed no significant difference in wound-healing rate. Healing rate before tamoxifen addition (μm h^−1^ ± s.e.m.) was: *CAGG-ERTM^Tg/−^ Vangl2^flox/flox^* 27.7 ± 4.1; *CAGG-ERTM^Tg/−^ Vangl2^flox/+^* 25.0 ± 2.5 (*n* = 6, 6). Tamoxifen addition did not affect the healing rate of the *CAGG-ERTM^Tg/−^ Vangl2^flox/+^* control cultures (compared with cells cultured without tamoxifen), but *Vangl2*-null cells healed significantly more slowly (figure 3*b*), confirming that loss of Vangl2 causes a planar migration phenotype. Healing rate after tamoxifen addition (μm h^−1^ ± s.e.m.) was: *CAGG-ERTM^Tg/−^ Vangl2^flox/flox^* 9.3 ± 2.4; *CAGG-ERTM^Tg/−^ Vangl2^flox/+^* 26.0 ± 7.2 (*n* = 8, 6; *p* = 0.0234).

PCP is mediated through pathways downstream of dishevelled that go through either DAAM1, RhoA and ROCK, or through JNK. Consistent with previous studies [25–27], it was found that inhibition of ROCK activity using Y-27632 increased the rate of healing of mouse corneal epithelial cells, whereas inhibition of JNK using SP600125 (as described in the Materials and methods section) decreased the rate of wound healing (mean ± s.e.m.; SP600125 7.99 µm h^−1^ ± 0.95; DMSO 14.13 µm h^−1^ ± 1.14; *t*-test: *p* = 0.001; *n* = 7; 8: Y-27632 26.06 ± 1.53; control 16.50 ± 2.02; *t*-test: *p* = 0.001; *n* = 11; 9; figure 3*c,d*). Previous data have suggested that increased wound-healing rate of epithelia upon inhibition of ROCK activity is substantially owing to cytoskeletal disruption causing cells to spread into the wound [26,27]. Labelling the actin cytoskeleton of corneal epithelial cells with Alexa488-conjugated phalloidin after *Vangl2*-deletion, inhibition of JNK and inhibition of ROCK activity was consistent with this possibility (figure 3*e–h*). Whereas control, *CAGG-ERTM^+^ Vangl2^flox/flox^* tamoxifen-treated epithelia and JNK-inhibited cells all showed a dense actin cytoskeleton with stress fibres and focal adhesions at the leading edge of the migrating epithelial sheet, ROCK-inhibited cells exhibited a less dense network, with near-absence of actin structure at the leading edge (figure 3*h*).

Together, these data show that Vangl2 is not absolutely required for wound healing, but the absence of Vangl2 causes a significant wound-healing delay. The data show that in the absence of Vangl2, cells still reorganize their cytoplasm normally to heal a wound. The migration and cytoskeletal consequences of *Vangl2* deletion are recapitulated by JNK inhibition and are in contrast to the accelerated migration and cytoskeletal abnormalities cause by ROCK inhibition. Hence, the wound-healing activity of the core PCP pathway is most likely mediated through JNK-induced changes to facilitate normal cell migration.

### 2.4. Core planar cell polarity pathway components control realignment of corneal epithelial cells in response to contact-mediated cues

Corneal epithelial cells have previously been shown to have a robust contact-mediated planar response: they align parallel to grooved substrata, with cytoskeletal reorganization effected by small GTPase activity [28]. In order to determine whether PCP pathways affect the alignment response to contact-mediated guidance cues, HCE cells were plated on quartz slides with parallel grooves 320 nm deep and 4 µm wide that mimic the dimensions of the corneal epithelial basement membrane [29], with or without genetic or pharmacological manipulation of PCP components, as described in Materials and methods.

**Figure 4.**
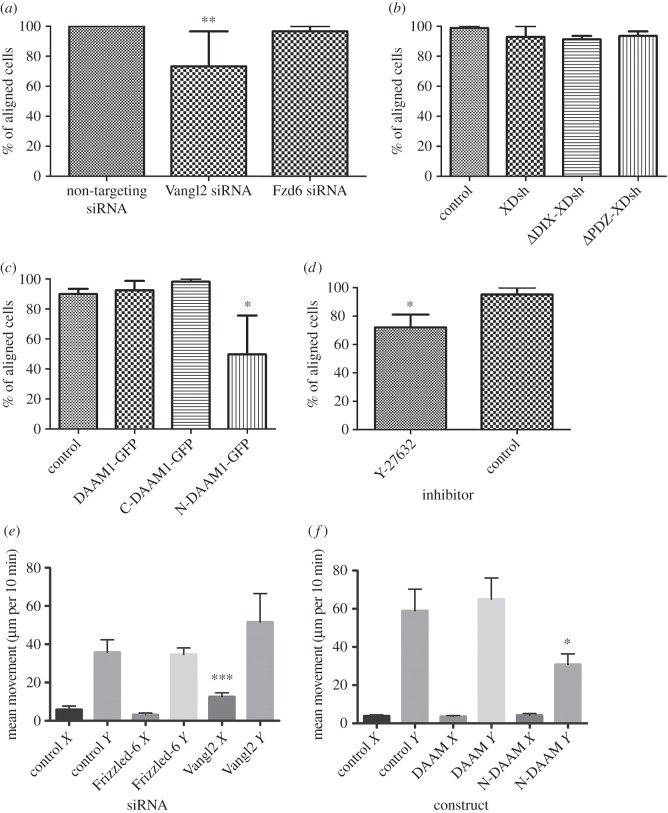
PCP components mediate planar alignment and migration of human corneal epithelial (HCE) cells on quartz nanogrooves. HCE cells were plated onto quartz slides with parallel grooves of rectangular profile, depth 320 nm, width 4 µm. Cell orientation and migration parallel to the grooves was measured after 24 h. (*a*) siRNA-mediated knockdown of VANGL2 to 30% of normal levels led to significantly reduced cell orientation parallel to the grooves compared with cells transfected with nonsense control siRNA after 24 h (*p* = 0.004), but knockdown of FZD6 to 35% of normal levels had no effect. (*b*) Transfection of HCE cells with constructs encoding wt Dvl2, or mutant forms of Dvl2 lacking the DIX or PDZ domains, caused no change in cell orientation at 24 h (*p* = 0.49) compared with ‘empty’ vector controls. (*c*) Transfection of HCE cells with constructs encoding wt DAAM1 or constitutively active C-DAAM1 caused no change in cell orientation at 24 h, but dominant-negative N-DAAM1 led to a significant reduction in cell orientation parallel to grooves (*p* < 0.05) compared with ‘empty’ vector controls. (*d*) Cells treated with ROCK-inhibitor Y-27632 showed significantly reduced orientation parallel to grooves after 24 h culture (*p* = 0.0005) compared with vehicle-treated cells. (*e*) Migration of cells (µm per 10 min) transfected with siRNA targeting VANGL2, FZD6 or nonsense non-targeting siRNA. There was no significant difference in migration parallel to grooves in any group (*p* = 0.31) but cells with Vangl2 knockdown showed a very significant tendency to migrate across grooves (*x*-direction, *p* = 0.0001) compared with controls. (*f*) Migration of cells transfected with constructs expressing wt DAAM1 or dominant-negative N-DAAM1 (µm per 10 min). Compared with cells transfected with empty vector control, neither DAAM1 construct affected migration across the grooves (*x*-axis, *p* = 0.84); however, the N-DAAM1 construct significantly impaired migration parallel to grooves (*y*-axis, *p* = 0.015).

HCE cells aligned their long axes almost perfectly to the grooves in control experiments. Cells transfected with siRNA targeted to *VANGL2* showed significantly less efficient alignment compared with those transfected with control, non-targeting, siRNA both 4 and 24 h after plating (% aligned ± s.e.m.: 4 h; VANGL2 63.3 ± 10; control 73.4 ± 11.9: *t*-test; *p* = 0.04; *n* = 3 experiments, more than 500 cells; 24 h; VANGL2 73.3 ± 23.3; control 100 ± 0: *t*-test; *p* = 0.004; *n* = 3 independent experiments, more than 500 cells; figure 4*a*). This showed that normal VANGL2 dosage is required for optimal contact-mediated cell alignment. Knockdown of *FZD6* did not produce any comparable defect in cell alignment—possibly because the degree of knockdown was insufficient and/or because of redundancy with FZD3.

Intracellular mediators of the PCP pathway were disrupted to determine their role in cell alignment. Plasmids constitutively expressing GFP-tagged alleles of dishevelled-2 were transfected into HCE cells: wild-type (Dsh-GFP); an allele lacking the PDZ domain that acts as a dominant-negative inhibitor of ROCK activation (ΔPDZ-Dsh-GFP); and an allele lacking the DIX domain that acts as a dominant-negative inhibitor of canonical Wnt signalling (ΔDIX-Dsh-GFP). GFP expression was used as a marker of successful transfection, and only fluorescent cells were assayed. However, none of these constructs had any significant effect, compared with ‘empty’ GFP controls, on HCE cell alignment either at 4 h (% of aligned cells ± s.e.m.; XDsh-GFP 92.9 ± 5.0; Δ-DIX-XDsh-GFP 91.2 ± 0.26; Δ-PDZ-XDsh-GFP 89.2 ± 1.5; control 98.9 ± 1.1; *n* = 3) or at 24 h (% of aligned cells; XDsh-GFP 92.9 ± 7.1; Δ-DIX-XDsh-GFP 91.2 ± 2.3; Δ-PDZ-XDsh-GFP 93.6 ± 3.1; control 98.8 ± 1.2; *n* = 3) (figure 4*b*). These data were not significantly different (4 h: one-way ANOVA, *F*_3,446_ = 0.7884, *p* = 0.5008; 24 h: *F*_3,198_ = 0.8126, *p* = 0.4882).

Although the data suggested that Vangl2 did not control cell alignment via a dishevelled-2-mediated pathway, it was considered possible that redundancy with dishevelled-1 and dishevelled-3 was masking a potential effect of dishevelled-2 manipulation on the downstream effector DAAM1. Hence, DAAM1 was manipulated directly. The alignment on grooves of HCE cells expressing GFP-tagged alleles of human DAAM1, constitutively active C-DAAM1 and dominant-negative N-DAAM1 were assayed. Inhibition of PCP with N-DAAM1 caused a significant loss of cell alignment, in contrast to cells overexpressing wild-type DAAM1 or C-DAAM1, for which cell alignment was not significantly affected (4 h: % aligned cells ± s.e.m.; control-GFP 91.4 ± 2.7; DAAM1-GFP 81.1 ± 6.4; C-DAAM1-GFP 100 ± 0; N-DAAM1-GFP 66.1 ± 9.2; *n* = 3; 24 h: % aligned cells; control-GFP 90.0 ± 3.5; DAAM1-GFP 92.4 ± 6.3; C-DAAM1-GFP 98.2 ± 1.8; N-DAAM1-GFP 49.7 ± 25.9; *n* = 3 independent experiments; figure 4*c*). One-way analysis of variance was performed on cosine-transformed data from all three of the DAAM1 constructs and the ‘empty’ GFP control and confirmed significance (*F*_3,416_ = 17.85, *p* < 0.0001). This was consistent with the possibility that Vangl2 mediated HCE cell alignment through dishevelled and DAAM1, and hence presumably through ROCK activity. Consequently, analysis of the alignment of the HCE cells in the presence of the ROCK inhibitor Y-27632 showed a significant reduction in the rate of ‘parallel’ realignment compared with controls (% aligned cells ± s.e.m.: 4 h; Y-27632 58.7 ± 9.4; control 95.8 ± 4.2: *t*-test; *p* = 0.012; *n* = 3; 24 h; ROCK 72.1 ± 9.0; control 95.2 ± 4.8: *t*-test; *p* = 0.0005; *n* = 3; figure 4*d*). Inhibition of JNK activity using inhibitor SP600125 caused no significant change in realignment compared with vehicle-treated controls (% aligned cells ± s.e.m.: 4 h; SP600125 98.5 ± 1.5; DMSO 88.1 ± 1.1: *t*-test; *p* = 0.188; *n* = 3; 24 h; SP600125 98.7 ± 1.3; DMSO 100 ± 0: *t*-test; *p* = 0.193; *n* = 3 independent experiments).

### 2.5. Normal VANGL2 dosage is required for correctly aligned migration on grooves

In addition to aligning parallel to grooved quartz slides, HCE cells also migrated up and down the grooves. HCE cells were therefore transfected with siRNAs targeting VANGL2 or FZD6, with controls as before, and their migration parallel to the grooves (*y*-axis) and across (perpendicular to) the grooves (*x*-axis) was measured using time-lapse microscopy. Cells transfected with the siRNA targeting VANGL2 showed no difference in their ability to migrate parallel to grooves, compared with cells transfected with the non-targeting control siRNA (one-way ANOVA, *y*-axis: *F*_2,154_ = 1.199; *p* = 0.3044), but had a significantly increased tendency to migrate across the grooves (one-way ANOVA, *x*-axis: *F*_2,154_ = 11.30; *p* < 0.0001; figure 4*e*). *Post hoc* comparison was performed using Tukey HSD test which showed that the mean movement of Vangl2 siRNA-treated cells along the *x*-axis, perpendicular to grooves (mean = 12.55, s.d. = 14.34) was significantly greater than cells targeted with siRNA against either Frizzled-6 (mean = 3.20, s.d. = 7.47) or control siRNA (mean = 5.81, s.d. = 10.76). Consistent with the alignment data above, siRNA-mediated knockdown of FZD6 had no significant effect on cell migration.

These data suggested that with depletion of VANGL2, cells were not sensing or responding correctly to grooves and that VANGL2 may be required for normal contact-mediated planar cell migration of these adult epithelial cells.

To determine whether the failure of cell alignment on grooves caused by expression of dominant-negative N-DAAM1 (figure 4*c*) was translated to defective contact-mediated planar cell migration, HCE cells were transfected with plasmids expressing DAAM1 and N-DAAM1, as well as empty vector controls as above. Neither DAAM1 nor dominant-negative N-DAAM1 had a significant effect on HCE cell migration perpendicular to grooves (one-way ANOVA: *F*_2,89_ = 0.1770, *p* = 0.8381). However, transfection with N-DAAM1 did significantly reduce migration parallel to the grooves (*F*_2,89_ = 4.397, *p* = 0.0151). Tukey HSD *post hoc* test showed that the mean movement of cells transfected with DAAM1 (mean = 64.97, s.d. = 66.62) and N-DAAM1 (mean = 30.79, s.d. = 34.30) differed significantly (*p* < 0.05; figure 4*f*).

### 2.6. Conditional knockout of Vangl2 ablates *in vivo* patterns of centripetal migration in corneal epithelia

**Figure 5.**
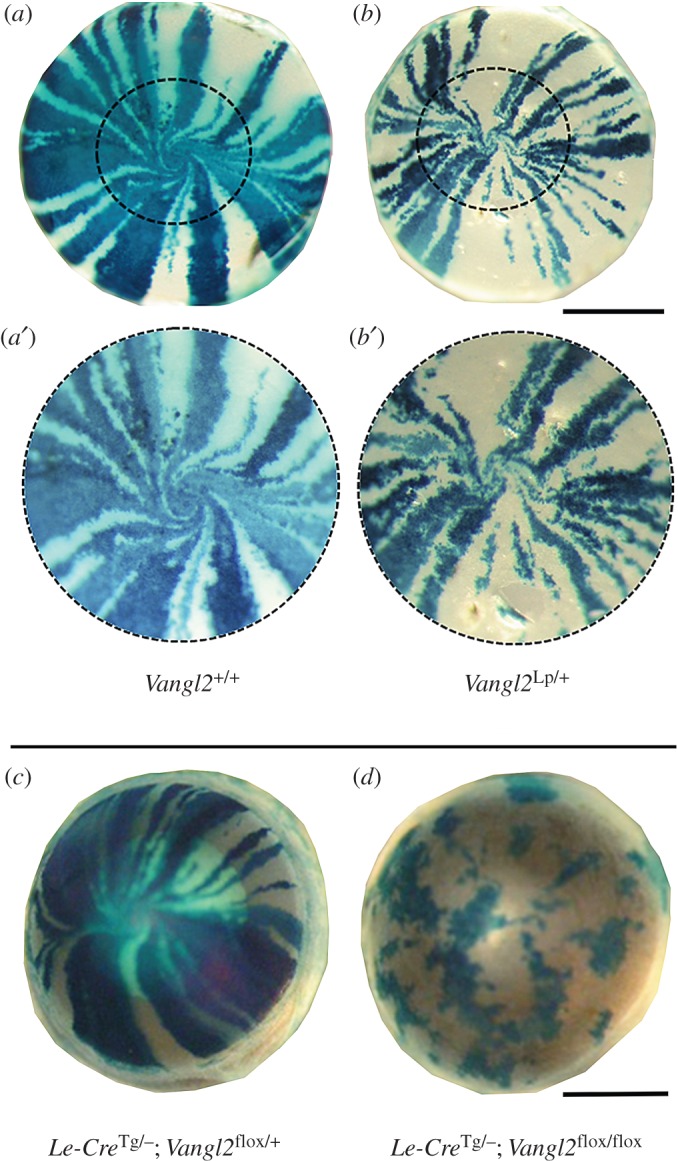
Patterns of cell migration in Vangl2-deficient corneal epithelia. (*a,b*) X-Gal staining of adult wild-type (*a* and inset *a*′) and *Vangl2^Lp/+^* (*b* and inset *b*′) littermates on the *XLacZ* reporter transgenic background. Inset *b*′ shows only mild, if any consistent disruption of tracks of migrating cells near the centre of the cornea where migrating cells converge. (*c,d*) Conditional deletion of *Vangl2* in the corneal epithelium. X-Gal staining of corneas of adult *Le-Cre^+^ Vangl2^flox/+^* mice (*c*) and disrupted patterns in their *Le-Cre^Tg/−^ Vangl2^flox/+^* littermates. Absence of striping indicates failure of normal patterns of centripetal cell migration. Scale bars, 1 mm.

Data presented above showed that core PCP components mediate planar cell alignment and migration in experimental situations. To test whether Vangl2/PCP mediates planar migration of adult corneal epithelial cells *in vivo* in a physically and pharmacologically unperturbed system, *Vangl2^Lp/+^* (Looptail) mice and conditional knockouts were used to assay the centripetal migration of corneal cells from the limbus that continues through adult life. These patterns of cell migration have previously been visualized using mice with mosaic expression of *LacZ* or fluorescent markers [3–5,8]. The H253 ‘XLacZ’ mouse has an X-linked *LacZ* transgene driven by a housekeeping promoter [30]. It exhibits regular radial stripes of *LacZ* expression in adult female mice owing to random X-inactivation during early embryonic life leading to clones of both *LacZ*-positive and *LacZ*-negative limbal stem cells coexisting at the corneal periphery. The *XLacZ* transgene was bred onto the *Vangl2^Lp/+^* background. Adult female *Vangl2^Lp/+^ XLacZ^Tg/^*^−^ mice showed striping patterns that were grossly normal (figure 5*a,b*). The estimated number of coherent clones of limbal stem cells in wild-type and mice (derived by counting stripes as described in the Materials and methods section) showed no significant difference between *Vangl2^Lp/+^* and *Vangl2^+/+^* mice (electronic supplementary material, figure S7). A mild disruption of striping was noted at the centre of the cornea of most (9/13) *Vangl2^Lp/+^ XLacZ^Tg/^*^−^ mice where, compared with their wild-type littermates, the streams of migrating cells appeared to break up and divert, with partial loss of the regular vortex-like patterns often observed in the normal corneal epithelium (figure 5*a′*,*b′*). This suggested that reduction of Vangl2 dosage caused only mild disruption of centripetal migration *in vivo*.

Transgenic mice, carrying the *Le-Cre* transgene that expresses *Cre* in the corneal epithelium throughout life, were bred to *Vangl2^flox/flox^* mice, and the *Le-Cre^Tg/−^ Vangl2^flox/+^* progeny was backcrossed to *Vangl2^flox/flox^* to produce *Le-Cre^Tg/−^ Vangl2^flox/flox^* mice and *Le-Cre^Tg/−^ Vangl2^flox/+^* controls. The *Le-Cre^Tg/−^*, *XLacZ*, *Vangl2^flox/+^* is a critical control because it has been shown that eye abnormalities can occur in *Le-Cre^Tg/−^* mice on some genetic backgrounds, even without a floxed target gene [31]. RT-PCR and immunohistochemistry confirmed that *Vangl2* was successfully deleted in *Le-Cre^Tg/−^ Vangl2^flox/flox^* corneal epithelia and no mRNA or protein was detected (electronic supplementary material, figure S8). The adult *Le-Cre^Tg/−^ Vangl2^flox/flox^* conditional knockouts had grossly normal eyes compared with *Le-Cre^Tg/−^ Vangl2^flox/+^* littermates, with transparent corneas, lens and retinal tissues apparently morphologically unchanged upon visual inspection. Expression of *Fzd6* was not affected (electronic supplementary material, figure S8).

When the *XLacZ* transgene was bred onto the *LeCre^Tg/−^ Vangl2^flox/flox^* background the *LeCre^Tg/−^*, *XLacZ*, *Vangl2^flox/+^* (*n* = 11) and all *LeCre^−/−^ XLacZ* (*n* = 31) female controls showed qualitatively normal patterns of radial striping at 12–15 weeks old, indicating the LeCre transgene by itself has no effect on striping. In contrast, the *LeCre^Tg/−^*, *XLacZ*, *Vangl2^flox/flox^* eyes (*n* = 8) had highly disrupted patterns of β-gal staining (figure 5*c,d*). Coherent clones of *LacZ^+^* cells were irregularly shaped and largely restricted within the corneal epithelium. The lack of stripes made an estimation of the number of stem cell clones impossible. These data were consistent with the hypothesis that normal centripetal migration would fail in the *Vangl2*-deleted corneal epithelium as a result of loss of core PCP activity and confirmed that Vangl2 was required for normal planar behaviour *in vivo*.

## 3. Discussion

PCP pathways have multiple roles during vertebrate embryogenesis, but little is known about the roles of core PCP genes once primary organogenesis is finished. PCP activity has been shown to modulate cancer cell invasion metastasis, but tumours themselves fundamentally recapitulate ‘embryonic’ systems [32]. It seems very likely that any epithelial system that has the capacity to repair and regenerate in the adult may use a PCP-based mechanism: postnatal airway epithelial cells display robust planar polarity, and fish lateral lines are another example of potential persistence of PCP into the adult [33,34]. However, there is little experimental evidence to elucidate the mechanisms of action. In this study, for the first time, we have demonstrated a role for the core PCP protein, Vangl2, in planar behaviour in a normal adult vertebrate system. The study has shown that that core PCP transmembrane protein Vangl2 is an essential component of the mechanism that corneal epithelial cells use to interpret guidance cues. The key results were:

(1) Multiple core PCP genes are expressed in the adult vertebrate corneal epithelium *in vivo*, including those encoding the transmembrane proteins Vangl2 and Fzd-6.
(2) Disruption of Vangl2 activity leads to loss of planar alignment of migrating corneal epithelial cells *in vitro* and *in vivo* in a dose-dependent manner.
(3) Core PCP function is required for interpretation of physical guidance cues that regulate cell alignment.

PCP signalling is mediated through the three homologues of the intracellular protein, dishevelled1–3. The DEP domain of dishevelled activates JNK pathways, and the PDZ domain mediates actin cytoskeletal rearrangement via DAAM1, RhoA and ROCK1/2. The defects in cell migration as a result of *Vangl2* disruption during wound healing could be mimicked by inhibiting JNK. In contrast, defects in cellular *alignment* (e.g. on grooved substrates) could in part be mimicked by inhibition of ROCK, the other major downstream effector. Tentatively, we propose a model suggesting that Vangl2-directed planar behaviour acts through the DEP domain of Dishevelled and JNK-induced cytoskeletal/motility changes [35] to mediate normal migration speed, and through the PDZ domain of Dishevelled, via DAAM1 and ROCK, to mediate normal cell alignment, at least in response to contact-mediated cues.

The Looptail mutation *Vangl2^Lp/+^* is known to act in a semi-dominant manner [36], and our data have shown that corneal epithelial cells are sensitive to dosage of *Vangl2*. Although heterozygous *Vangl2^Lp/+^* corneas showed only mild defects of radial migration of epithelial cells, assayed on the basis of disruption of radial stripes in female *XLacZ* reporter mice, deletion of *Vangl2* resulted in very severe disruption. The eyes of *Vangl2^Lp/+^* mice were grossly normal, and although mild defects of cell migration were noted on the *XLacZ* background, wound-healing rate of *Vangl2^Lp/+^* epithelial cells was not significantly different from wild-type. However, corneal epithelial cells with conditional deletion of *Vangl2* showed a very significant delay in wound healing. Cells in which VANGL2 activity was reduced to around 30% of normal by targeting siRNA showed mild defects in the alignment and cell migration *in vitro*. This is in contrast with the data for its putative binding partner, Frizzled-6, for which no migration defect was observed in knockdown cells. This is consistent with the recessive nature of Fzd6 mutation [37] and may be because of redundancy with Frizzled-3, which was also found to be expressed in the adult corneal epithelium, but not studied further here. Whereas Vangl1 may cooperate with Vangl2 during embryonic development, it cannot compensate for Vangl2, and deletion of *Vangl2* ablates all fzPCP activity [38].

A wound-healing defect or abnormal pattern of cell migration *in vivo* may be explicable by factors other than abnormalities of cellular planar alignment. However, our *in vitro* data showing that genetic or pharmacological disruption of PCP activity reduced the ability of HCE cells to align on grooved substrata and to migrate cathodally in an applied electric field confirmed a direct proximal effect of disruption of PCP on planar behaviour.

Disruption or knockout of Vangl2 pathways did not lead to failure of cell migration *per se*, only its directionality. In applied electric fields, speed of siRNA-mediated Vangl2-knockdown cells was not significantly different from that of controls, but they took a more wandering migration track. Similarly, scratch-wound assays on Vangl2-deleted mouse corneal epithelial cells did not prevent wound healing, but caused a significant delay. We also pharmacologically inhibited two downstream mediators of PCP signalling, ROCK and JNK, and found that neither prevented wound healing. Consistent with previous data [27], ROCK inhibition increased the rate of wound healing, associated with disruption of the cytoskeleton leading to apparent stretching of cells behind the wound edge. Together with the observation that the actin cytoskeleton was not grossly defective in Vangl2-deleted corneal epithelial cells, these data suggest that reorganization of the actin cytoskeleton (the primary role of ROCK in this system) is not the main mechanism through which PCP acts during the wound-healing migration response.

Manipulation of Fzd-6 by itself produced no directional migration abnormalities in the *in vitro* systems used here. There are several potential explanations. *Fzd6^−/−^* mice are viable and fertile and show only mild PCP defects [37], so the level of siRNA-mediated knockdown in this study may have been insufficient. Related to the above possibility is the likely redundancy with other PCP components including Fzd-3. A final exciting possibility is that the directional phenomena explored here are mediated through a different receptor such as the receptor-like tyrosine kinase, Ryk [39]. Polarized migration of cells within the developing limb, similar to the movement of corneal epithelial cells observed here, is Frizzled-independent and instead requires ROR2 [40]. These possibilities need to be investigated further.

In this study, HCE cells were aligned on grooved quartz surfaces whose dimensions mimic those of the basement membrane extracellular matrix upon which the cells normally migrate [28,41]. However, the biological relevance of this observation requires further explanation. Scanning electron microscopy has previously shown that both basement membrane extracellular matrix and the underlying stromal collagen do not show any overt non-random orientation with respect to the centre of the cornea [41]. It is therefore difficult to see how they could provide a contact-mediated guidance cue during centripetal corneal epithelial cell migration. Our parallel study [42] has shown however that HCE cells may receive contact-mediated alignment from the underlying basement membrane, suggesting some cryptic underlying asymmetry that epithelial cells can read. If cells do receive a contact-mediated centripetal cue, then they must be able to override it during wound-healing cell migration, as off-centre wounds cause epithelial cells to form a new centre of convergence [7].

### 3.1. Failure of striping patterns of cell migration in Vangl2-knockout corneas

Radial patterns of mosaic transgene activity in the corneal epithelium of normal adult mice are owing to centripetal migration of cells from the limbus towards the centre of the cornea [3–5]. In previous studies, LESC activity and epithelial cell migration were not detectable until mice were several weeks old, and at birth, a more random patchwork pattern of mosaic transgene activity was observed [3,7,41]. Persistence of this disrupted, patchwork patterning into the adult animal, as observed in *Vangl2*-knockout corneas in this study, has been shown previously to correlate with failure of limbal stem cell activity and/or of directed cell migration in the cornea (reviewed by Mort *et al.* [7]). For example, in *Dstn^corn1/corn1^*, CAGG-EGFP mice, with a destrin deletion, mosaic GFP expression and severe corneal epithelial abnormalities, cell migration fails during adult life leading to globular disorganized mosaic patterns of GFP expression [43]. Mutations in the gene encoding the ocular surface transcription factor, Pax6, which is required for alignment of corneal epithelial cells on grooved substrates, also lead to disrupted, patchy mosaic patterns [42,44,45]. Of note, in these studies, clones of corneal epithelial cells appear to remain largely coherent even when cell migration has stopped or is disrupted. This supports a model of highly proliferative blue and white basal epithelial cells maintaining coherent patches of epithelium such that totally mixed salt-and-pepper patterns of single cells are not normally observed—as was the case in this study for the Vangl2-knockout corneas.

The absence of radial stripes from *LeCre^Tg/−^*, *XLacZ*, *Vangl2^flox/flox^* corneas could therefore be explained in several ways. Vangl2 may be required for limbal stem cell specification or function, or for the transition from neonatal, limbal-independent mode of corneal maintenance to adult, limbal-dependent maintenance. These possibilities would imply the Vangl2-knockout corneal epithelia are maintained without limbal stem cell input, either because the stem cells are not present or not functional. A level of total LESC failure is unlikely as this would be expected to lead to severe ocular surface disease [46] not observed in any of the multiple knockout eyes here. It was shown in this study that loss of Vangl2 does not prevent corneal epithelial cells from migrating, but affects directionality, so the most likely explanation is a failure of planar behaviour. A significant directional cue for corneal epithelial cells is likely to be the step change in substrate compliance at the limbal corneal boundary [47,48]. Epithelial cells exhibiting durotactic movement from regions of high-to-low substrate compliance are likely to move from the limbal to the corneal epithelium, and this may be sufficient to initiate centripetal movement across the ocular surface [42,47,49]. That the patterns of XGal staining in the Vangl2-knockout cornea are consistent with failure of centripetal migration may suggest that Vangl2 is required for proper interpretation or directional response to the changes in rigidity of the substrate at the limbal corneal boundary. Alternatively, we have recently shown that corneal epithelial cells receive a component of radial directionality from an interaction with the basement membrane [42] and Vangl2 may be crucial for interpretation of these contact-mediated cues. If either of the above scenarios is true, then the disorganized patches of XGal staining in the Vangl2-knockout corneas represent combinations of probably coherent clones formed when cells proliferate to maintain the corneal epithelium as superficial cells are shed. Regardless of the explanation for the absence of radial stripes, it is clear that they do not form normally and that Vangl2 is critical for normal corneal planar epithelial movement.

## 4. Material and methods

### 4.1. Mice

Hemizygous *Le-Cre^Tg/−^* mice (*Tg(Pax6-cre,GFP)1Pgr*), driving expression of *Cre* recombinase in the lens and corneal epithelia [50] and *CAGG-CreERTM^Tg/−^* mice (*Tg*(*CAG-cre/Esr1*)) driving constitutive expression of tamoxifen-inducible *Cre* [51,52] were bred with *Vangl2^flox/flox^* mice [24] and H253 (‘XLacZ’) reporter mice carrying X-linked *nLacZ* under the control of a housekeeping promoter [30]. *Vangl2^Lp/+^* mice were as described in [36]. All mice were maintained on the CBA/Ca genetic background. Genomic DNA was obtained through digestion of ear clips samples using proteinase K (10 mg ml^−1^) and clean-up using the QIAamp DNA mini kit (Qiagen, Manchester, UK). PCR mixtures were made up as a 50 µl reaction mixture: 30 µl dd H_2_O; 5 µl 5× buffer Y (Peqlab UK); 5 µl of forward and reverse primers (10 mM); 2 µl dNTP mix; 0.5 µl Taq DNA polymerase (Peqlab UK) and 1 µl of digested DNA sample. Genomic amplification performed on DNA from *Vangl2^flox/+^* animals with *Vangl2* primers (F: 5′-CCGCTGGCTTTCCTGCTGCTG-3′ and R: 5′-TCCTCGCCATCCCACCCTCTG-3′) with 35 cycles, touchdown annealing from 67.5 to 60°C produced two possible amplicons of 701 bp (floxed allele) and 560 bp (wild-type allele). The presence of *Cre* was determined by using primers F 5′-TGCCCAAGAACCTTGATGGAC-3′ and R 5-CCCAGAAATCCCCAGATTAC-3 (35 cycles, annealing 59°C) that lie within the Cre transgene producing an amplicon of 430 bp. *XLacZ* mice were genotyped for the presence of the *LacZ* transgene (F: 5′-ATGAACGGTCTGGTCTTTGC-3′ and R: 5′-ACATCCAGAGGCACTTCACC-3′), 35 cycles, 50°C annealing.

### 4.2. Reverse transcription polymerase chain reaction

RT-PCR was performed to investigate gene expression in cells and tissues on RNA isolated using the PEQGOLD Total RNA Kit (Peqlab, Southampton, UK) according to the manufacturer's instructions. For preparation of cDNA, the following were added to a nuclease-free microcentrifuge tube: Oligo(dT)_12–18_ (500 µl ml^−1^) 1 µl; 8 µl of RNA sample; 1 µl dNTP mix (10 mM) and sterile water up to 12 µl. The mixture was heated to 65°C for 5 min and then chilled on ice. 4 µl of 5× first strand buffer, 2 µl 0.1 M dithiothreitol and 1 µl RNase inhibitor (Promega, UK) were added. After incubating at 42°C for 2 min, 200 units of SuperScript II RT (Invitrogen, UK) were added and incubated at 42°C for 50 min. The reaction was then inactivated by heating to 70°C for 15 min and diluted to the appropriate concentration of 6 µg µl^−1^. Primers and expected band sizes are given in table 1 and electronic supplementary material, table S1.

**Table 1.** Primer sequences used to detect expression of PCP genes in mouse corneal epithelia.

gene	sequence 5′ to 3′	product length (bp)
*Vangl2-*F	TGAGGGCCTCTTCATCTCC	535
*Vangl2-*R	ACCAATAACTCCACGGG	
*Frizzled-6-*F	GAACTAGCACAGGAGCGACC	463
*Frizzled-6-*R	AGTTTTCTTCAAGCGTCGGA	
*Dishevelled1-F*	CCTGGGACTACCTCCAGACA	502
*Dishevelled1-R*	CTTGGCCACTGTGAGACTGA	
*Dishevelled2-F*	TTTGCAGGTAAATGATATGA	497
*Dishevelled2-R*	ACAGCCCACTGGCATACTTG	
*Dishevelled3-F*	TTCGGGAGATTGTGCACAAG	459
*Dishevelled3-R*	GCAGGTTGCTAGCATACTTG	
*Daam-1-F*	TGCACTTCAGACAATGGAGC	416
*Daam-1-R*	ACTCAGAGGTCTCATAGTCC	
*RhoA-F*	CTTTATAAGTGATGGCTGCC	501
*RhoA-R*	TGGTCTTTGCTGAACACTCC	
*Rock1-F*	CAAGCTTGAAGAGCAACTGC	505
*Rock1-R*	CTTGTCTGCTTGTGACTTGG	
*Rock2-F*	AGAACACCTTAGCAGTGAGG	412
*Rock2-R*	GGAACTTTCTGCCTGGG	

### 4.3. Real-time quantitative polymerase chain reaction

Real-time amplification was performed in 384 well plates using a LightCycler 480 real-time PCR machine (Roche). Primers for quantitative PCR (qPCR) assay of PCP gene expression were designed using the online Roche Universal Probe Library Assay Design Centre (http://lifescience.roche.com/shop/en/us/overviews/brand/universal-probe-library; electronic supplementary material, table S2).

### 4.4. Cell culture

The adult HCE cell line (HCE-S) [53] was maintained in DMEM/F12 medium with 10% fetal calf serum. Plasmids encoding GFP-tagged wild-type and mutant forms of human DAAM1, dominant-negative hN-DAAM1 and constitutively active hC-DAAM1 [54] were a kind gift from Raymond Habas (Temple University, PA). Amaxa nucleofection was performed on 1 × 10^6^ cells using 5 µg of DNA following the manufacturer's protocol T-020 for epithelial cells. For gene knockdown in HCE cells, siRNAs targeting human *VANGL2* and *FZD6* were obtained from Qiagen (Manchester, UK; electronic supplementary material, table S3). 6 × 10^4^ cells were seeded into a 24-well plate with 0.1 ml of medium. 100 µl of 10 nM siRNA (diluted in serum free media) was added to 3 µl of HiPerFect transfection reagent (Qiagen, UK) and mixed by vortexing for 10 s. This mixture was incubated at room temperature for 10 min before being added dropwise to the wells containing the cells. Cells were then incubated at 37°C/5% CO_2_ for 3 h before 400 µl of culture medium was added to each well. Cells were then incubated as before for 48 h before being used in experiments described below. Efficacy of knockdown was confirmed by qPCR at Aberdeen University core facility. ‘AllStars’ negative control siRNA (Qiagen), with no homology to any human gene, was transfected in parallel to *Vangl2* and *Frizzled6* targeting siRNAs as a negative control. ‘AllStars’ Hs Cell Death Control siRNA (Qiagen) served as positive control to ensure successful (more than 90%) transfection of the siRNA through detection of cell death. The Hs cell death control is a mixture of siRNAs that knocks down multiple anti-apoptotic genes.

Primary mouse corneal epithelial cell culture was performed as described in [23,55]. Adult mice eyes were obtained at eight weeks old and stored in phosphate-buffered saline (PBS) at 4°C. Corneas were dissected and halved and placed into penicillin and streptomycin solution for 10 min. Each corneal half was then attached—epithelial face up—into a 35 mm tissues culture dish. Primary culture medium [55] was added. The cultures were kept at 37°C/5% CO_2_ and grown for 10 days before the corneal explant was removed. The confluent cultures were grown until three weeks old and then used in the experiments. Monolayer of cells *in vitro* was gently scratched using a 20 µl pipette tip without damaging the tissue culture dish. Wound width was recorded at 0, 2, 4, 6 and 24 h time points. Cells were kept at 37°C and 5% CO_2_ during healing.

### 4.5. Analysis of cell alignment and migration on grooved substrata

Grooved substrata were previously prepared by Department of Electronics and Engineering, Glasgow University, using electron beam lithography on fused quartz microscope slides as previously described [56]. The slides had 5 mm × 5 mm areas of parallel grooves—each of the 320 nm depth with width 4 µm. The areas of grooves were separated by areas of flat slide providing an internal control environment. Slides were sterilized in concentrated nitric acid overnight followed by three rinses with sterile PBS and 10 min UV exposure. HCE cells were plated onto grooved slides, and images were obtained of the cells both 4 and 24 h later using a Leica DRIMB inverted microscope with temperature-controlled chamber and time-lapse video capture. Only successfully transfected GFP-positive cells were assayed. MTrack analysis software for ImageJ (http://imagej.nih.gov/ij/) was used to track cell migration in the *x-* and *y*-coordinates on grooved and control substrates, and for measuring the angles of the long axes of the cells in relation to the vertical grooves (*θ*). Cells plated on the ungrooved areas were measured, using a vertical line as the reference. Cells were binned with alignment 0–9°, 10–19°, 20–29°, relative to the grooves and distribution analysed following [28,57,58]. Several hundred cells were counted under each experimental condition. Statistical analysis was performed using GraphPad Prism.

### 4.6. Cell migration in applied electric fields

Twenty-four hours after gene silencing with siRNAs, HCE cells were exposed to physiological electric field stimulation to measure directionality of electrotactic movement. Cells were seeded into ‘ibidi’ 15 µm chamber-slides (Thistle Scientific) and time-lapse video recording performed, using a Nikon Diaphot inverted phase contrast microscope with a temperature-controlled environment chamber. HCE cells were subjected to direct current electric field by adopting a set-up previously described by Rajnicek *et al.* [58]. Agar-salt bridges were used to connect silver/silver chloride electrodes in beakers of Steinberg's solution. HCE cells were exposed to either no electric field (no-EF) or EF strengths of 100 mV mm^−1^ and 200 mV mm^−1^ for a period of 3 h. FMIX, directionality and speed of movement were measured by using the ‘manual tracking’ and ‘chemotaxis’ plugins (available at http://rsbweb.nih.gov/ij/plugins/index.html) for ImageJ. FMIX is one of the most indicative measures of migration and is determined by recording the migration track of the cells and dividing the distance moved along the *x*-axis (parallel to the applied EF, towards the cathode) by the total distance migrated (accumulated distanced, *d*). FMIX evaluates the magnitude of the directional cell movement towards the cathode. Conventionally, the cathode is at the left, so the closer is the FMIX value to −1 the more directed is the cell to the cathode on average. ‘Directionality’ was also measured as a parameter for studying the cellular dynamics upon application of an electric field. Directionality is given as Euclidian distance migrated (i.e. the length of the straight line connecting the start and endpoint of the cell's migration)/accumulated distance migrated. The closer is the directionality value to 1, the more directly the cell is moving between the start and endpoint. Directionality → 1: straight motion.

### 4.7. Immunocytochemistry

Cell monolayers were fixed with 4% paraformaldehyde in PBS for 10 min then washed three times in PBS, 2 min each. Blocking buffer (0.3% bovine serum albumin (BSA) in PBS, 4% serum, 0.1% Triton X-100) was added for 20 min. Primary antibody was added, diluted in blocking buffer, and left overnight at 4°C. Cells were washed three times in PBS for 2 min before the secondary antibody was added in blocking buffer for 1.5 h at room temperature. Cells were washed in PBS twice for 2 min. Coverslips were then placed over the cells using mounting medium (Vectashield, Vector Laboratories, UK) with DAPI nuclear dye. Table 2 lists all antibodies used and their complimentary secondary antibodies. Negative controls were performed with no primary antibody.

**Table 2.** List of antibodies used in the ICC and IHC experiments.

	host	supplier	dilution
primary antibody			
IgG anti-Frizzled-6	goat	Santa Cruz	1 : 250
IgG anti-hFrizzled-6	goat	R&D (AF3149)	1 : 200
IgG anti Vangl2	rabbit	Santa Cruz	1 : 250
secondary antibody DAB IHC			
anti-rabbit IgG biotinylated	goat	Sigma Life Science 851M6241	1 : 200
anti-mouse IgG1 Alexa 488	goat	Invitrogen (A21121)	1 : 250
anti-rabbit IgG Alexa 594	chicken	Invitrogen (A21442)	1 : 250
anti-goat IgG	donkey	Invitrogen (A11055)	1 : 250

### 4.8. Immunohistochemistry

Eyes were fixed in 4% paraformaldehyde in PBS, 2 h, washed three times in PBS, dehydrated through an ethanol series, placed in xylene overnight and wax-embedded. Sections (7 µm) were cut, dewaxed with Histoclear and rehydrated through an ethanol series into PBS, and endogenous peroxidases were quenched using 3% H_2_O_2_ in PBS (15 min). After two washes in PBS, antigen retrieval was performed by 20 min boiling in 0.01 M sodium citrate pH 6, followed by washing in PBS. Primary and secondary antibody incubations were performed, then sections were stained with haematoxylin, dehydrated and mounted with coverslips in DPX medium.

### 4.9. Wholemount cornea immunostaining

Eyes were collected from eight week old mice and fixed in 4% paraformaldehyde in PBS for 2 h followed by three washes in PBS. Eyes were then incubated in methanol for 2 h at 4°C and washed three times in PBS for 10 min. The cornea was then dissected and digested in 1% pepsin, 10 mM HCl, for 10 min at 37°C, then neutralized in 0.1 M sodium borate (pH 8.5) to stop pepsin activity. Corneas were washed in PBS twice, 10 min, and blocking buffer (as above) was added for 30 min at room temperature. Primary antibody was then added diluted in blocking buffer, overnight at 4°C. Corneas were washed in PBS, and secondary antibody was added diluted in the blocking buffer at room temperature for 3 h. The corneas were washed three times in PBS for an hour and flat-mounted in Vectashield with DAPI nuclear stain.

### 4.10. X-Gal staining of adult corneas

Patterns of β-galactosidase activity were observed in whole corneas of *XLacZ^Tg/−^* hemizygous female animals after staining with 5-bromo-4-chloro-3-indolyl-β-d-galactopyranoside (X-Gal) substrate. Eyeballs were enucleated from 15 to 20 week old mice and rinsed briefly in PBS. Eyes were placed in fix solution for 1 h at 4°C (2% formaldehyde (Sigma), 0.2% gluteraldehyde (Sigma), 2 mM MgCl_2_, 5 mM EGTA in 0.1 M phosphate buffer pH 7.4) and then washed three times for 20 min with in excess of detergent wash (0.01% sodium deoxycholate (Sigma, Life Sciences), 0.02% Nonidet P40 (NP40 substitute, BioChemika, Fluka 74 385), 0.05% BSA (Sigma) in 0.1 M phosphate buffer). Eyes were then placed in X-Gal staining solution (25 ml detergent wash, 0.085% NaCl, 5 mM K_3_Fe(CN)_6_, 5 mM K_4_Fe(CN)_6_, 0.1% X-Gal dissolved in 0.5 ml dimethyl formamide (Sigma)) overnight at 37°C. After X-Gal staining, eyes were fixed in 4% paraformaldehyde for 1 h and washed with PBS before taking them up through an ethanol series (1 × 50%, 2 × 70% for 20 min each) and stored at 4°C. Corneas were dissected from the eyes as previously described, and images were obtained under a stereomicroscope with the cornea facing uppermost.

## Supplementary Material

Word document with supplementary figures and details of primers and siRNAs.

## Data accessibility

In addition to the supplementary data section supporting this article, datasets underlying the histograms are available from the Dryad digital repository: http://dx.doi.org/10.5061/dryad.1qh4c [59].
