# Supplementary material for: The core planar cell polarity gene, Vangl2, directs adult corneal epithelial cell alignment and migration
Source: R Soc Open Sci. 2016 Oct 19;3(10):160658. doi: 10.1098/rsos.160658 (PMC5099008; doi:10.1098/rsos.160658)
Supplement: Word document with supplementary figures and details of primers and siRNAs. [file rsos160658supp1.docx]

**A planar cell polarity pathway directs adult corneal epithelial cell alignment and migration.**

Amy S. Findlay*, D. Alessio Panzica*, Petr Walczysko, Amy B. Holt, Deborah J. Henderson^1^, John D. West^2^, Ann M. Rajnicek & J. Martin Collinson†

**Supplementary Data**

**Figure S1: Expression of core PCP genes in human corneal epithelial cells.**

**
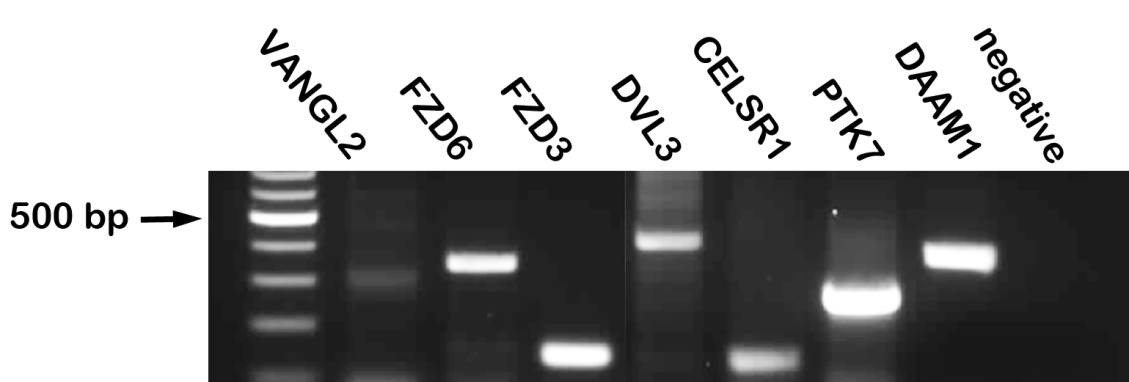
**

**Expression of core PCP genes in human corneal epithelial cells.** Total RNA was isolated from human corneal epithelial cells (HCE-S), reverse-transcribed as described in the Methods section, and RT-PCR performed using primers and annealing temperature described in Supplementary Table 1. (Duplicate lane removed from image).

**Figure S2: Expression of Fat-PCP genes in the adult mouse cornea.**

**
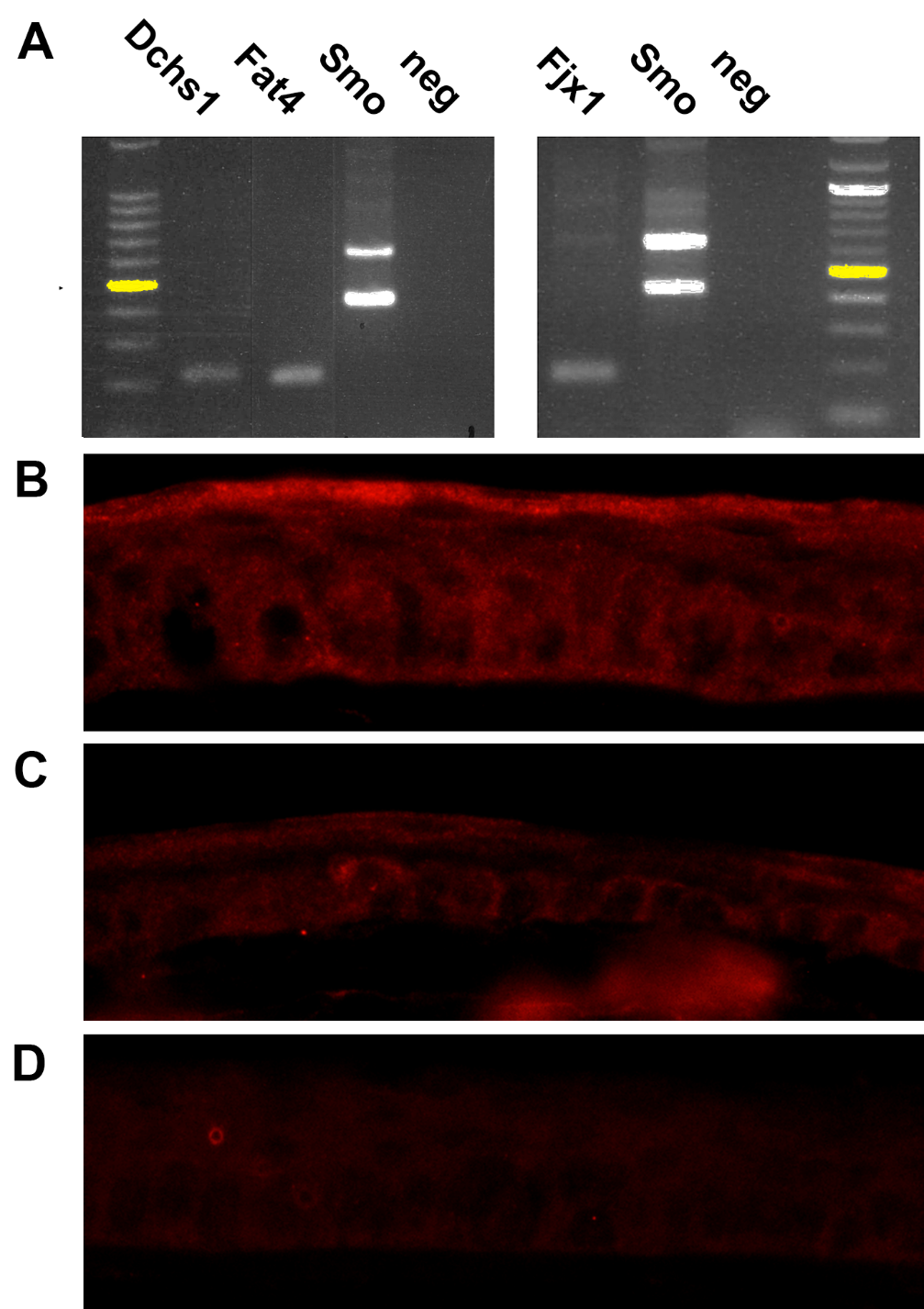
**

**Expression of Fat-PCP genes in the adult mouse cornea. (A)** RT-PCR on cDNA prepared from adult mouse corneal epithelia using primers for *Dachsous1* (*Dchs1*), *Fat4* and *Four-joint*ed 1 (*Fjx1*). The gene encoding the Hedgehog ligand co-receptor protein Smoothened was used as a positive control. Negative control included no cDNA. Yellow bands in ladders = 500 bp. Primers and expected band sizes are included in **Supplementary Table S2.** Duplicate lanes have been removed from the image. **(B)** Immunohistochemistry on tissue section of adult wild-type corneal epithelium (central region) using validated antibody against Fat4. **(C)** Image of Fat4 staining in limbal epithelium – same section as (B). **(D)** Negative control (no primary antibody).

Fat4 was expressed at low levels but there was some evidence of a gradient (limbal low to central high).

**Figure S3: Validation of siRNA silencing of VANGL2 in human corneal epithelial cells.**


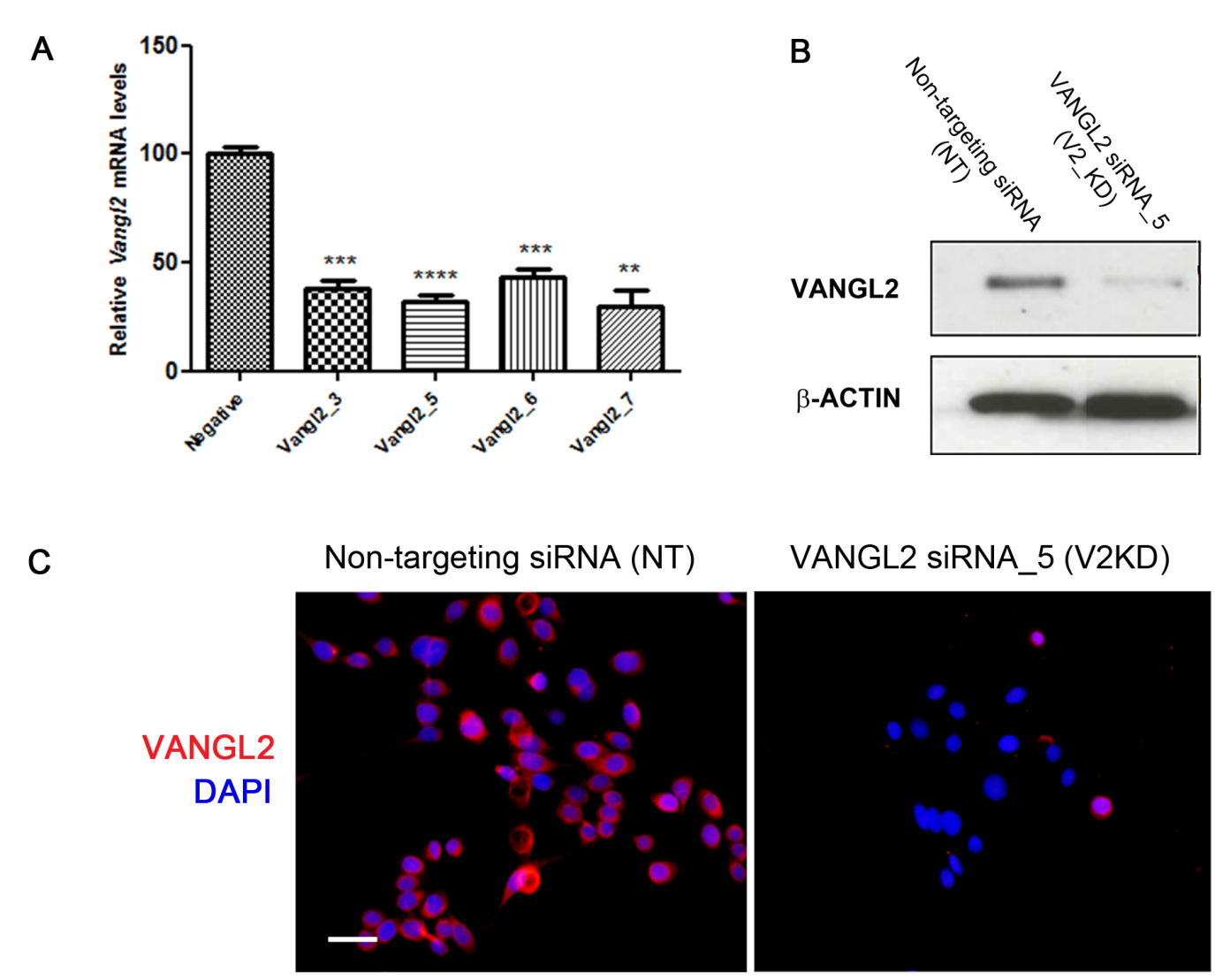


**Validation of siRNA silencing of VANGL2 in human corneal epithelial cells.** **(A)** Four unvalidated siRNAs targeted against human *VANGL2* were obtained from QIAGEN (Manchester, UK) (Vangl2_3, 5, 6, 7 – sequences in **Supplementary Table S4**), and transfected separately into human corneal epithelial cells as described in the Methods section. cDNA was made from cells transfected with each siRNA, and those transfected with a control non-targeting nonsense siRNA, and qPCR was performed using primers specific to human VANGL2 **(Supplementary Table S3)**. siRNA Vangl2_5 was shown to produce the most robust, consistent knockdown of *VANGL2* to 32% of wild-type levels )(mean of three experiments), and this siRNA was used exclusively for all further work described in the paper. **(B)** Western blot of protein lysate from human corneal epithelial cells transfected with siRNA Vangl2_5 or non-targeting siRNA, probed with antibodies against VANGL2 and β-actin confirming successful knockdown. (Intervening lanes removed from image). **(C)** Immunocytochemistry on human corneal epithelial cells transfected with siRNA Vangl2_5 or non-targeting siRNA, probed with antibody against VANGL2 (red), and labelled with DAPI nuclear stain (blue). ** = *P* < 0.01; *** = *P* < 0.001; **** = *P* < 0.0001.

**Figure S4: Validation of siRNA silencing of FZD6 in human corneal epithelial cells.**

**
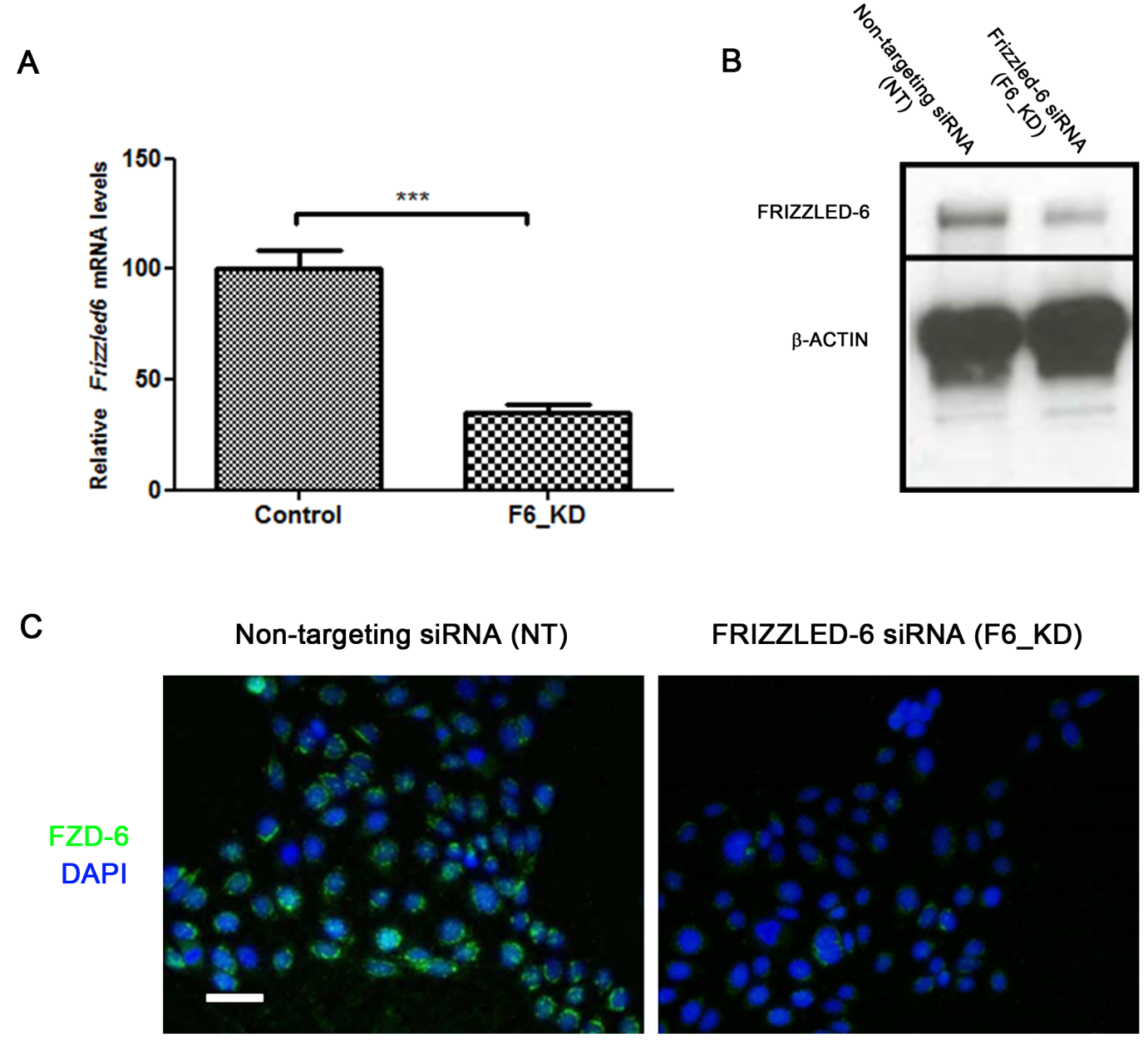
**

**Validation of siRNA silencing of FZD6 in human corneal epithelial cells.** **(A)** Previously validated siRNA targeted against human *FZD6* were obtained from QIAGEN (Manchester, UK) (sequences in **Supplementary Table S4**), and transfected into human corneal epithelial cells as described in the Methods section. cDNA was made from cells transfected with siRNA F6_KD, and those transfected with a control non-targeting nonsense siRNA, and qPCR was performed using primers specific to human *FZD6* **(Supplementary Table S3)**. siRNA F6_KD was shown to produce a robust knockdown of *VANGL2* to around 35% of wild-type levels (mean of three experiments). **(B)** Western blot of protein lysate from human corneal epithelial cells transfected with siRNA F6_KD or non-targeting siRNA, probed with antibodies against FZD6 and β-actin confirming successful knockdown. **(C)** Immunocytochemistry on human corneal epithelial cells transfected with siRNA F6_KD or non-targeting siRNA, probed with antibody against FZD6 (green), and labelled with DAPI nuclear stain (blue). *** = *P* < 0.001.

**Figure S5: Migration of human corneal epithelial cells in an applied physiological electric field.**

**A.**


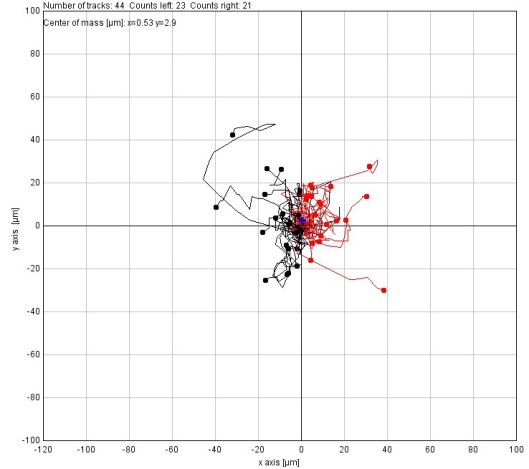

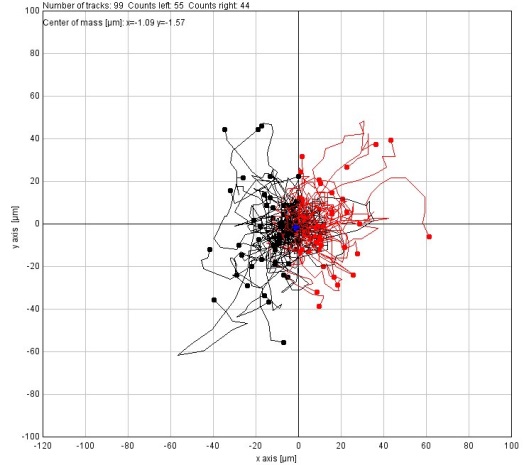

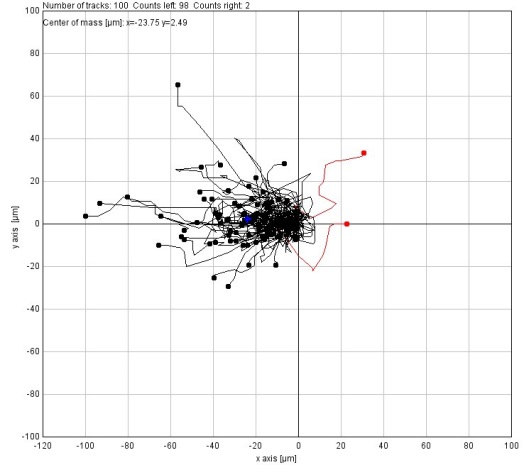

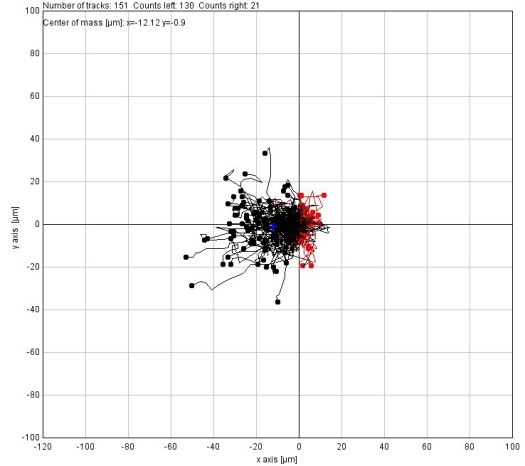


**Left**

**Right**

**Left**

**Right**

**Cathode**

**Anode**

**Cathode**

**Anode**

**μm**

**μm**

**μm**

**μm**

**μm**

**μm**

**μm**

**μm**

**Non-targeting siRNA control (NT)**

***Vangl2* targeting siRNA (*V2*_KD)**

**Non-targeting siRNA control (NT)**

**0 mV/mm**

**0 mV/mm**

**200 mV/mm**

**200 mV/mm**

**1.**

**2.**

**3.**

**4.**

***Vangl2* targeting siRNA (*V2*_KD)**


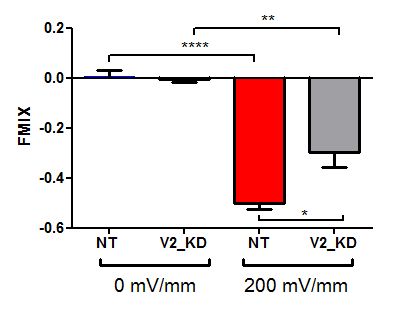

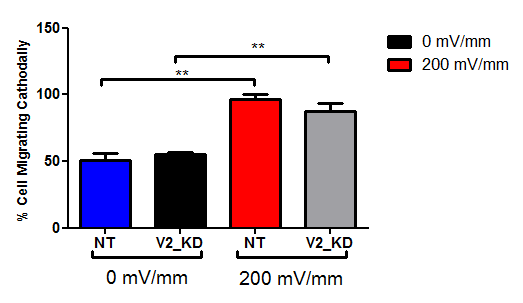


**B.**

**C.**

**Figure Legend overleaf.**

**Migration of human corneal epithelial cells in an applied physiological electric field.**

**(A**) ‘Spider’ drawings showing the migration paths taken by human corneal epithelial cells tracked in the experiment in presence or absence of an applied electric field. They are generated by using the cell tracking plugin of ImageJ at the different 18 time points over the 3 hour electric field exposure. ‘ibidi’ μ-Slides were placed in the incubation chamber of the microscope such that the cathode was to the left of the visual field. The track all cells treated in the same way (field or no-field, *VANGL2*-knockdown or nonsense siRNA) were overlaid on the appropriate plot. Plot drawings are presented on the same scale, with each square in the grid = 20 μm. Each graph contains a number of cells represented by dots of a different colour, red if the final destination of a cell is to the anodal side (right) of its starting point meaning that the cell has migrated towards the anode and vice-versa for the black dots. Migration paths are represented by lines relative to their point of origin. Similar numbers of red and black dots in 0 mV/mm experimental conditions display the random migration of the cells. In contrast the greater number of cells represented with black dots in the 200 mV/mm electric field demonstrates cathodal migration. Dashed circle in (A) 4. delineates an area of where multiple cells migrated cathodally a short distance such that their individual tracks are not distinguishable.

**(B, C)** As described in the main text, the forward migration index (FMIX) is lower in *V2*_KD cells indicating their less efficient cathodal migration compared to the NT control cells, as suggested by the spider plots. Negative values indicate that the movement was towards the cathode (on the left side of the μ-Slide). Extent of cathodal displacement over the 3 h time-lapse experiment is represented as percentage of cells migrating cathodally in **C.** Without EF exposure approximately the 50% of both NT and *V2*_KD cells migrated cathodally, indicating random cell movement. A high proportion of cathodal movement is observed when cells were exposed to 200 mV/mm EF field strength. *t*-tests: *, p < 0.05; **, p < 0.01; ****, p < 0.0001. A slightly smaller proportion of cells treated with *VANGL2*-targeting siRNA moved cathodally after application of electric field, compared to controls, but the difference was not significant. However the cells that moved cathodally did so less directly and therefore did not travel as far towards the cathode on average (see spider plots and main Results section. The data show that VANGL2 is not required to sense the applied electric field, but is required for robust directional migration.

**Figure S6: Tamoxifen-inducible deletion of *Vangl2* in mouse corneal epithelial cells**


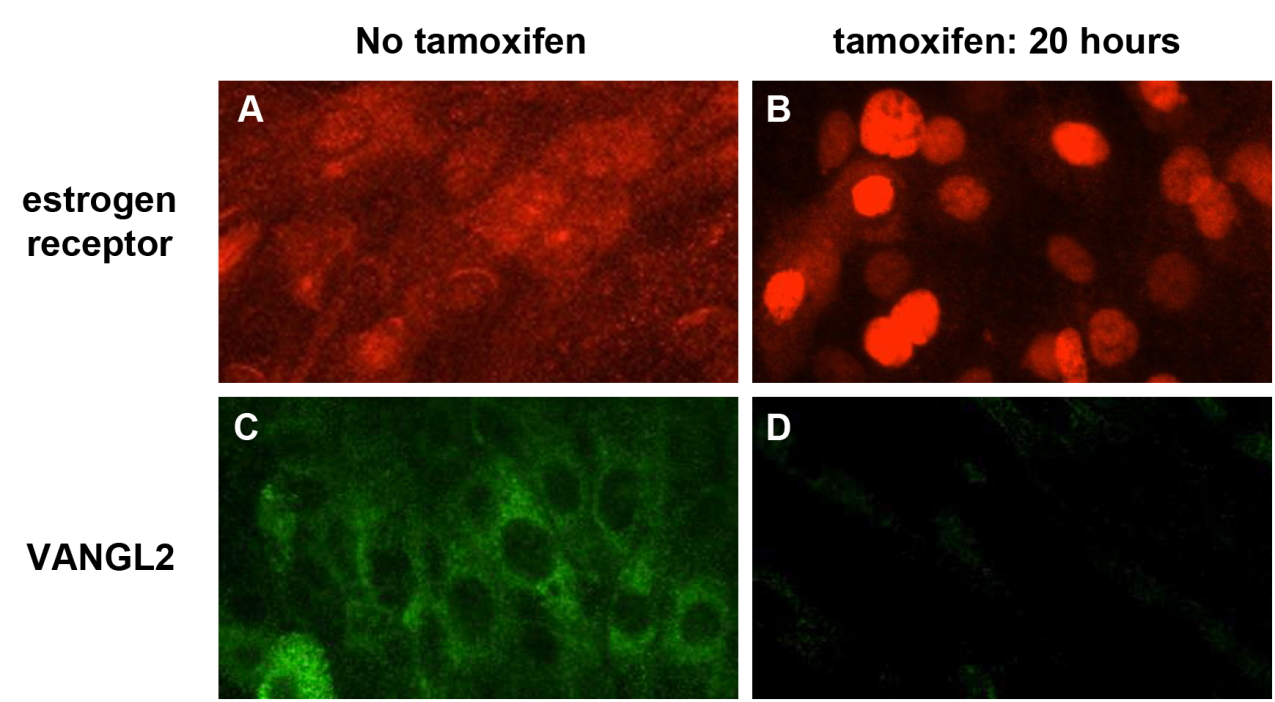


**Tamoxifen-inducible deletion of Vangl2 in mouse corneal epithelial cells. (A-D)** Cultures of CAGG-*CreERTM^Tg/+^ Vangl2^flox/flox^* mouse corneal epithelial cells immunostained for the estrogen receptor ERα (A, B) (red) or Vangl2 (C, D) (green) in control media (A, C) or after 20 hours of exposure to 4-OH tamoxifen (B, D) as described in main text. Addition of tamoxifen leads to almost complete loss of Vangl2 within 24 hours. The anti-ERα antibody recognises both endogenous estrogen receptor and the mutant tamoxifen-sensitive Cre-ERTM, hence there is both nuclear and cytoplasmic staining in control conditions, but shows clear nuclear relocalisation upon addition of tamoxifen.

**Figure S7: Estimation of number of coherent clones of limbal epithelial stem cells maintaining *Vangl2^Lp/+^* corneal epithelia.**

**
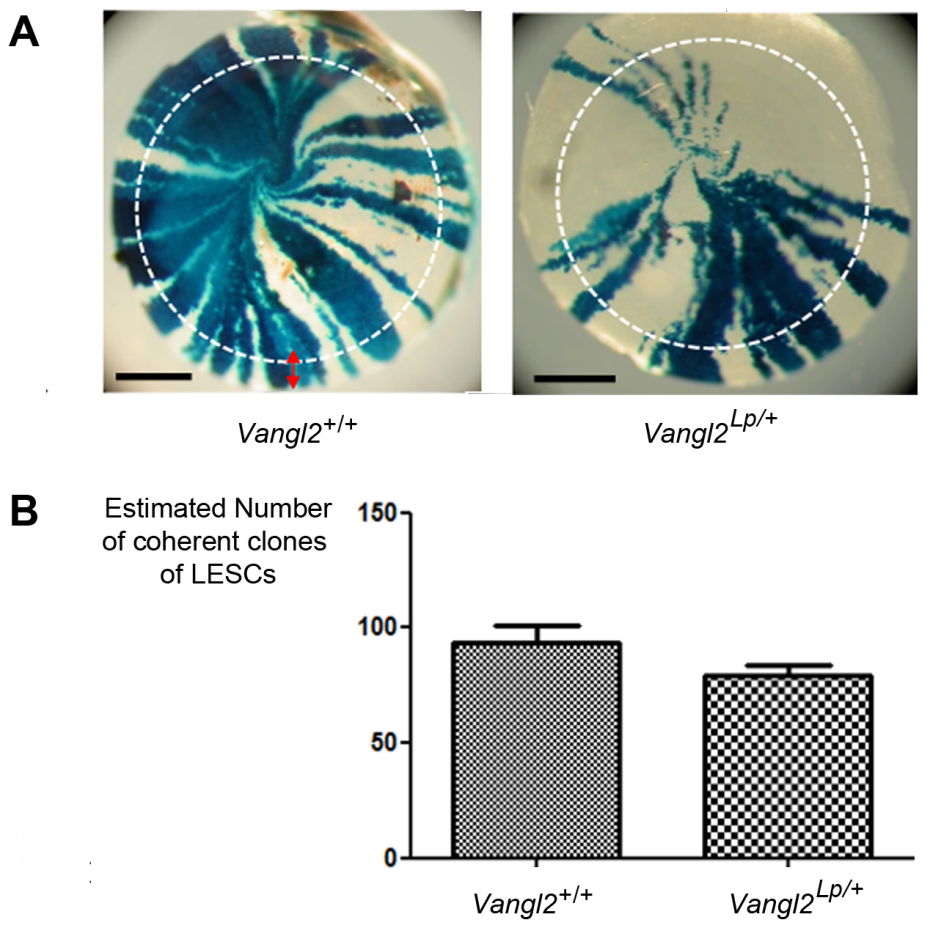
**

**Estimation of number of coherent clones of limbal epithelial stem cells maintaining *Vangl2^Lp/+^* corneal epithelia.** The evaluation of the self-renewal capacity of the corneal epithelium was based on the estimation of the number of independent coherent clones of stem cells at the limbus. Each *LacZ^+^* (blue) and *LacZ^-^* (white) stripe is derived from a blue or white patch of limbal epithelial stem cells respectively. Each blue or white patch of limbal epithelial stem cells is in turn composed of one or more clones of blue or white stem cells, each clone descended from a single blue or white stem cell specified during development. The greater the proportion of the corneal epithelial that is blue (P), the greater the probability that each blue patch is composed of more than one clone of blue stem cells. To correct for this, the following two assumptions are made: 1) that any clone does not split into two, and 2) that clone sizes are normally distributed. By counting the number of blue stripes transected by a circle drawn round the periphery of the corneal epithelium and multiplying by the correction 1/(1-P) an estimation of the number of coherent clones of limal epithelial stem cells can be made for each cornea (2) (West, 1976).

**(A)** Representative examples of *Vangl2^+/+^* and *Vangl2^Lp/+^* corneas showing peripheral circles transecting the stripes. **(B)** Estimated number of independent coherent clones of limbal epithelial stem cells trended downward between between *Vangl2^+/+^* (93.50 ± 7.47; n = 12) and *Vangl2^Lp/+^* (79.46 ± 3.91; n = 16) animals but the difference was not significant (Unpaired *t*-test: *P* = 0.0861). Comparison was made between littermates 12-15 weeks old.

West, J. D. 1976. Clonal development of the retinal epithelium in mouse chimaeras and X-inactivation mosaics. *J. Embryol. Exp. Morph.* **35**: 445-461.

**Supplementary Figure S8: Validation of corneal epithelial *Vangl2* deletion by the *LeCre* transgene**


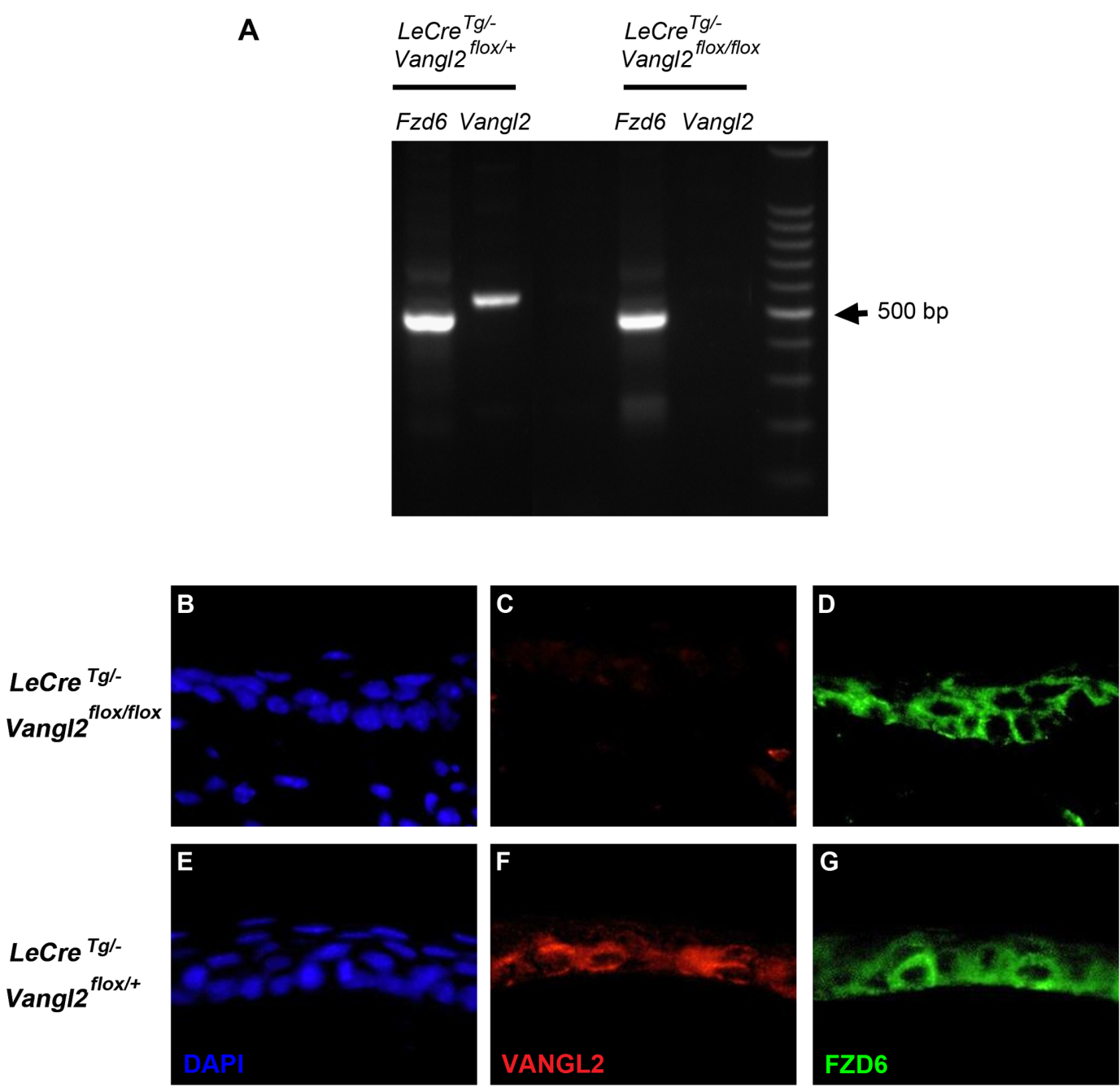


**Validation of corneal epithelial *Vangl2* deletion by the *LeCre* transgene. (A)** RT-PCR on cDNA prepared from corneal epithelia of adult *LeCre^Tg/-^ Vangl2^flox/+^* and *LeCre^Tg/-^ Vangl2^flox/flox^* littermates. Primers were as described in Table 1 – the Vangl2 primers were designed within exon 4 of Vangl2 which is deleted by Cre. *Fzd6* was expressed in both genotypes but *Vangl2* expression was not detectable in corneal epithelia from *LeCre^Tg/-^ Vangl2^flox/flox^* mice. (B-G) Immunofluorescence on tissue sections of corneas of *LeCre^Tg/-^ Vangl2^flox/+^* and *LeCre^Tg/-^ Vangl2^flox/flox^* littermates, showing DAPI nuclear staining (B, E), Vangl2 immunostaining (C, F) and Fzd6 immunostaining (D, G). Vangl2 was undetectable in corneal epithelia of the conditional knockouts but Frizzled-6 was unaffected.

**Supplementary Table S1:**

Primer sequences and expected band sizes for RT-PCR of genes encoding PCP components in human corneal epithelial cells (see **Supplementary Fig. S1**).

| **Gene** | **Sequence 5’ to 3’** | **Product length (bp)** |
| --- | --- | --- |
| *CELSR1* F | TCATCGCCAACAATGGCACCC | 163 |
| *CELSR1* R | TGAGGCATGGCTTGCTCACAG |  |
| *DAAM1* F | GCACCACTGCCTCCAAATGCCT | 421 |
| *DAAM 1* R | ACAGACGGCCCTCCTGCTGAG |  |
| *DISHEVELED3* F | GATGGGCAGGAGACGCCGTA | 444 |
| *DISHEVELED 3* R | TAGCCGGGTTGCATGCTCTGG |  |
| *FRIZZLED3* F | GCCCCCTTTGCCCAAACCCA | 154 |
| *FRIZZLED3* R | AGGATGCTTCAGGCATTGCGCTT |  |
| *FRIZZLED6* F | CGGAGCTAGCACCCCCAGGT | 154 |
| *FRIZZLED6* R | TGGACTAATCCTTCCTTCACTCCGC |  |
| *VANGL22* F | AGGAGGGCCAGGCTTGTAGTGG | 309 |
| *VANGL2* R | GGCCAGCCAGCGTTCCTTGT |  |
| *PTK7* F | AAGTGGGAACGGGCAGATGGGAG | 303 |
| *PTK7* R | GGTGGGGTCCAGGATGCGGT |  |

**Supplementary Table S2:**

Primer sequences and expected band sizes for RT-PCR of genes encoding Fat-PCP components in mouse corneal epithelial cells (see **Supplementay Fig. S2**).

| **Gene** | **Sequence 5’ to 3’** | **Product length (bp)** |
| --- | --- | --- |
| Fat4F |  |  |
| *Fat4 R* | TGAGGCATGGCTTGCTCACAG |  |
| *Dchs1* F | GCACCACTGCCTCCAAATGCCT | 421 |
| *Dchs1* R | ACAGACGGCCCTCCTGCTGAG |  |
| *Fjx1* F | GATGGGCAGGAGACGCCGTA | 444 |
| *Fjx1* R | TAGCCGGGTTGCATGCTCTGG |  |
| *Smo* F | GCCCCCTTTGCCCAAACCCA | 154 |
| *Smo* R | AGGATGCTTCAGGCATTGCGCTT |  |

## Supplementary Table 3. PCR oligonucleotide primers used in the real time qPCR of human corneal epithelial cells.

| Gene name | Sequence 5’ to 3’ | Amplicon length (nt) |
| --- | --- | --- |
| *Daam-1-*F | GGAGCTACAAGTTGGCCTGA | 83 |
| *Daam-1-*R | TCCTTCTCTAAAGCCAGCAGA |  |
| *Frizzled-3-*F | ACAGCAAAGTGAGCAGCTACC | 73 |
| *Frizzled-3-*R | CTGTAACTGCAGGGCGTGTA |  |
| *Frizzled-6-*F | CGTCTATGAGCAAGTGAACAGG | 109 |
| *Frizzled-6-*R | CTTCGACTTTCACTGATTGGACT |  |
| *Vangl2-*F | GCCAGCCGCTTCTACAAC | 64 |
| *Vangl2-*R | TCTCCAGGATCCACACTGC |  |
| *GAPDH-*F  *GAPDH-*R | AGAAGGCTGGGGCTCATTTG  AGGGGCCATCCACAGTCTTC | 173 |

**Supplementary Table S4**

siRNA target sequences used for the silencing of *Vangl2* and *Frizzled-6* in human corneal epithelial cells. (See **Supplementary Figs. S3 and S4**).

| siRNA | Target sequence 5’-3’ |
| --- | --- |
| *VANGL2_3* | AUGGCAAACCCUGAUGAUGUA |
| *VANGL2_5* | UAGAAUUAGGAAGUACCCAUA |
| *VANGL2_6* | CCGGAGCGCUGCGGAUACAAA |
| *VANGL2_7* | CAGGCUUGUAGUGGCGGUGGA |
| *FRIZZLED6* | AGCGAUAUUAGUGCCAAUCAA |
